# Reinforced-count simulation for decision calibration under over-dispersed multi-type service demand

**DOI:** 10.1371/journal.pone.0358767

**Published:** 2026-09-18

**Authors:** Saisai Hou, Yunzhi Zhu, Sen Zhang, Ying Chen

**Affiliations:** 1 Department of Public Basic Courses, Nanjing University of Industry Technology, Nanjing, China; 2 Office of Academic Affairs, Nanjing Medical University, Nanjing, China; Northwest Normal University, CHINA

## Abstract

Service counts are often converted into capacity or inventory decisions with independent Poisson models, although clustering and latent heterogeneity can make the counts substantially more variable. We present reinforced-count simulation (RCS) as a low-parameter, pre-deployment stress test: it asks whether a decision calibrated under independence remains adequate when type shares persist. RCS is compared with independent Poisson, negative-binomial, empirical-residual and Scarf moment-robust decisions. The empirical analysis uses two public datasets. RAND Health Insurance Experiment physician-visit counts provide a cross-sectional held-out test (20,190 observations), and five categories of New York City 311 requests provide an external temporal test (1,096 days and 26 rolling origins). In the RAND analysis at a lost-event-to-holding-cost ratio of 20, over-dispersion-aware decisions reduced held-out cost relative to Poisson by 15.0% for RCS, 15.6% for negative binomial and 17.3% for the empirical quantile; fill rate increased from 76.5% to 88.2%–91.1%. In the NYC analysis at a ratio of 10, the RCS mean paired cost improvement was 18.7% (95% confidence interval, 12.4%–25.0%) and fill rate increased from 93.3% to 96.8%. A multi-type transfer experiment showed that each calibrated model was best in its matching environment; using a mismatched model produced 6.0%–20.0% regret. Joint sensitivity analysis, concentration-parameter learning curves and cost-ratio perturbations identify when the diagnostic is useful and when parameter error can dominate model choice. RCS is a reproducible check on mean-based decisions when composition dependence is uncertain, not a general demand model.

## Introduction

Count data drive many operational decisions. Outpatient visits, call-center contacts, maintenance requests, spare-parts failures and public service requests are translated into staffing, capacity, stock or replenishment levels. Independent Poisson or fixed-share multinomial models are common starting points because they are transparent and easy to calibrate. They can nevertheless be too concentrated when demand is affected by persistent users, shared shocks, latent rate variation or temporary changes in the composition of request types.

The practical question is not only which distribution fits best. A model matters when it changes an action. If a thin-tailed model selects too little capacity, the relevant loss is the resulting shortage or service failure, not the misspecified variance by itself. This distinction has long been central to inventory and newsvendor research [1–8], coordinated replenishment [9–11], call-center operations [12–14], emergency-service planning [15,16], and predictive and distributionally robust decision making [17–20]. Intermittent inventory demand poses a related model-selection problem [21].

Several familiar models address extra-Poisson variation. Negative-binomial and mixed-Poisson models capture latent rate heterogeneity [22–24]; Conway–Maxwell–Poisson regression accommodates both over- and under-dispersion [25]; copulas can represent dependence across margins [26]; and modern forecasting methods can learn nonlinear temporal structure [27]. Reinforced urn models supply a compact mechanism in which early random differences in type shares persist, producing heterogeneous compositions even when the baseline mean vector is fixed. Reinforcement connects classical exchangeability to modern self-exciting count processes [28–32].

We use this mechanism to stress-test decisions under persistent type shares. The classical moment identities are known [33–37]. Their operational value is that they expose a diagnostic blind spot. For cumulative type count $D_{i}(m)$ after *m* events, with baseline share $p_{i}$ and concentration θ,


$$\begin{array}{c} \mathrm{Var}\{D_{i}(m)\}=p_{i}(1-p_{i})\frac{m(m+\theta )}{\theta +1}, \end{array}$$ (1)

$$\begin{array}{c} \mathrm{Cov}\{D_{i}(m),D_{j}(m)\}=-p_{i}p_{j}\frac{m(m+\theta )}{\theta +1},\;i\neq j. \end{array}$$ (2)

Relative to independent multinomial sampling, both expressions contain the same inflation factor (m+θ)/(θ+1). The resulting normalized cumulative-count correlation does not depend on θ. A correlation screen can therefore miss a change in dispersion that matters to tail exposure and resource allocation. This invariance applies to the classical balanced urn; empirical count processes may behave differently.

We evaluate the diagnostic with a cross-sectional RAND-HIE split, 26 rolling origins of NYC 311 requests, a Scarf moment-robust comparator, a multi-type policy-transfer experiment and a joint κ×γ sensitivity grid. Concentration-learning and cost-ratio experiments further examine when model uncertainty changes the selected action. Together, these analyses address scalar and coordinated decisions, cross-sectional and temporal validation, and uncertainty in both the count mechanism and the economic inputs.

The contribution is a reproducible pre-deployment calibration workflow for the intermediate situation in which analysts can estimate exposure and mean demand and observe extra-Poisson variation, yet cannot identify a rich multivariate dependence model with confidence.

## Materials and methods

### Study design and diagnostic workflow

**Fig 1:**
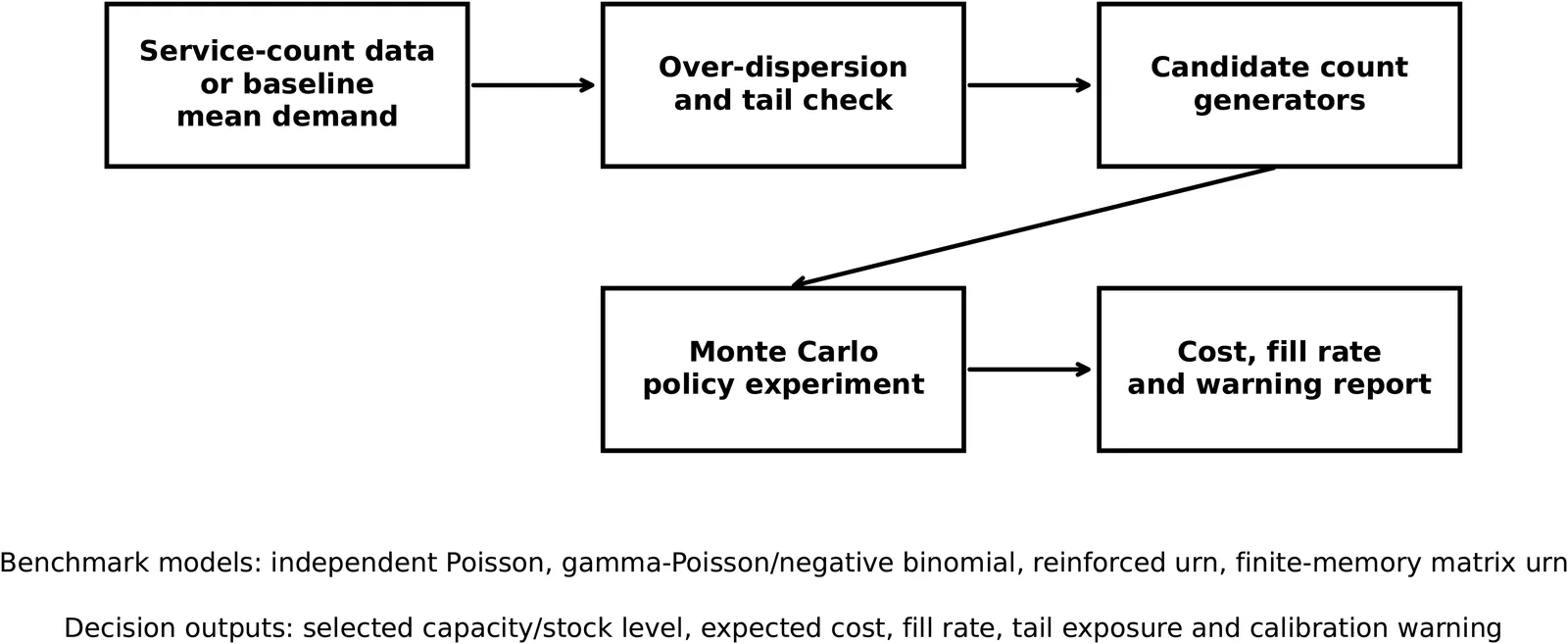
Reinforced-count simulation workflow. Observed counts and exposure information are used to estimate predictable means and diagnose residual dispersion. Candidate count generators then select actions that are evaluated on a common held-out or simulated demand environment. The output is a calibration warning, not a claim that the reinforced generator is the true model.

The workflow in Fig 1 separates three tasks: estimating the predictable mean, representing unresolved variation around that mean, and evaluating the decision induced by each representation. The same held-out observations are used to score all candidate decisions. A calibration warning is issued when the independence-selected action increases expected cost by at least 10% or reduces fill rate by at least five percentage points relative to an over-dispersion-aware alternative. These thresholds are reporting conventions that practitioners may replace with application-specific tolerances.

The computational sequence is: (1) define event types, exposure and decision costs; (2) estimate baseline means and inspect dispersion, tails and temporal stability; (3) construct independent, mixed-count, reinforced and robust benchmarks; (4) calibrate the same decision rule under each benchmark; (5) score all actions on common held-out observations or common random-number simulations; and (6) report cost, fill rate, regret, uncertainty and runtime.

### Public datasets

#### RAND-HIE physician visits.

The cross-sectional analysis uses the public, de-identified randhie dataset distributed with statsmodels [38–40]. It contains 20,190 annual counts of outpatient visits to a medical doctor. We group observations by self-rated health status (excellent, good, fair and poor). The variance-to-mean ratio, zero proportion and 95th percentile are calculated within each group. The authors had no access to direct or indirect identifiers. The stratified held-out experiment evaluates decision transfer within the sampled population; temporal validation is provided separately by the NYC analysis.

For each of 200 deterministic, stratified 50/50 splits, model parameters and candidate stock levels are obtained from the training half and scored on the disjoint test half. Four scalar decisions are compared: independent Poisson, negative-binomial, reinforced count (with a beta-Poisson scalar margin) and the empirical training quantile. Results are aggregated with health-group weights. Because RAND-HIE is cross-sectional, this experiment measures held-out decision transfer within the sampled population.

#### NYC 311 service requests.

External temporal validation uses NYC Open Data’s *311 Service Requests from 2020 to Present* dataset (identifier erm2-nwe9) [41]. We queried daily counts from 1 January 2022–31 December 2024 for five categories chosen before model evaluation: Illegal Parking, Noise – Residential, Blocked Driveway, Street Condition and Water System. The resulting panel has 1,096 days and 5,480 category-day counts with no missing cells. The submitted reproducibility package contains both the fixed aggregate snapshot and the exact Socrata download script. No address, location or personal field is retained.

For each category, expected daily demand is estimated by a Poisson generalized linear model with day-of-week and month indicators and a linear training-window trend. The role of this model is to remove predictable calendar structure before the residual count generator is calibrated; it is not presented as a state-of-the-art forecasting model. Each rolling origin uses the preceding 365 days for calibration and the next 28 days for evaluation. Origins are advanced by 28 days, producing 26 non-overlapping test windows from January 2023 through December 2024.

### Candidate count and decision models

Let $\mu_{t}=(\mu_{t1},...,\mu_{tK})$ be the estimated mean vector for decision period *t* and $\Lambda_{t}=\sum_{i}\mu_{ti}$. The candidate models intentionally share *t*his mean forecast and differ in their treatment of unresolved variation.

**Independent Poisson.** Counts are conditionally independent with $D_{ti}\sim \mathrm{Poisson}(\mu_{ti})$.

**Negative binomial.** Each type follows a gamma-Poisson mixture with variance $\mu_{ti}+\alpha_{i}\mu_{ti}^{2}$. The dispersion $\alpha_{i}$ is estimated from training residuals by moments. In the scalar RAND experiment, this model and the reinforced predictive can select similar decisions because both capture the relevant marginal over-dispersion; they are not distributionally identical.

**Reinforced count.** For each period,


$$\begin{array}{c} V_{t}\sim \mathrm{Dirichlet}(\theta p_{t}),\;D_{ti}|V_{t}\sim \mathrm{Poisson}(\Lambda_{t}V_{ti}), \end{array}$$

where $p_{ti}=\mu_{ti}/\Lambda_{t}$. Smaller θ produces more variable and persistent compositions; ρ=1/(θ+1) is a convenient reinforcement index. Conditional Poisson sampling preserves a fluctuating total as well as composition uncertainty.

**Empirical residual.** A training residual ratio is sampled jointly across types and multiplied by the forecast mean. This nonparametric benchmark preserves observed cross-type residual patterns within the training window.

**Scarf moment-DRO.** A distributionally robust stock level is selected using only the estimated mean and variance, minimizing the worst-case one-period holding and lost-event cost over distributions with those moments [19]. This classical comparator is distinct from modern distributionally robust optimization (DRO) methods based on Wasserstein or other data-driven ambiguity sets [20].

### Decision loss and evaluation measures

For demand *d*, capacity or stock *s*, holding cost *h* and lost-event cost *q*, the one-period loss is


$$\begin{array}{c} L(s,d)=h(s-d{)}_{+}+q(d-s{)}_{+}. \end{array}$$

The experiments set *h* = 1 and report q/h∈{5,10} for temporal validation, with additional values 2 and 20 in sensitivity analysis. Each model selects the smallest integer quantile corresponding to *q*/(*q* + *h*), or the robust counterpart for Scarf’s model. Fill rate is ∑min(D,S)/∑D. Regret is the percentage cost increase relative to the best candidate action in the stated test environment. For the rolling-origin analysis, paired percentage improvement is calculated within each origin before averaging; 95% confidence intervals use the standard error across the 26 origins.

For the multi-type experiment, a coordinated can-order rule (*s*,*c*,*S*) is evaluated. When any type reaches *s*, all types at or below *c* are replenished to *S*. Cost includes holding, lost events and a fixed setup charge $K_{f}=18$ per replenishment occasion; there is no per-unit order charge. This design makes the source of any coordination advantage explicit. Fill rate and setup frequency are reported with cost.

### Concentration calibration and data sufficiency

When a multi-type count sample is available, θ is estimated by maximum likelihood under the Dirichlet-multinomial composition model after conditioning on each period’s total count. If $y_{t}$ is the observed vector and $p_{t}$ the fitted baseline share, the optimized log likelihood is


$$\begin{array}{c} \ell (\theta )=\sum_{t}\left[\log\Gamma (\theta )-\log\Gamma (\theta +n_{t})+\sum_{i}\{\log\Gamma (\theta p_{ti}+y_{ti})-\log\Gamma (\theta p_{ti})\}\right]. \end{array}$$

Optimization is performed on logθ over [0.05,5000]. A simulation study evaluates estimation and capacity stability for true θ∈{3,10,30} and samples of 14, 28, 56, 112 and 224 periods, with 60 replications per condition. When only mean demand is available, θ is not identifiable. In that case the workflow reports a break-even grid: the largest reinforcement concentration at which the recommended integer decision first differs from the independent decision. The grid is a sensitivity range; estimating θ requires repeated count observations.

### Additional robustness experiments

Four experiments address model and parameter boundaries. First, 28-day windows of NYC counts are summarized by median variance-to-mean ratio, mean absolute cross-category correlation and median 95th-percentile-to-mean ratio. Their association is evaluated with Spearman correlation to illustrate how dispersion and correlation can move separately in observed counts.

Second, a finite-memory matrix urn is evaluated on a 6×6 grid with cross-share κ∈{0,0.10,0.25,0.40,0.60,0.80} and memory γ∈{0.90,0.95,0.97,0.985,0.995,1.00}. Each cell uses 140 replications of 760 periods after a 300-period warm-up. Dispersion, lag-one autocorrelation, pairwise correlation, cost, fill rate and coordination advantage are retained. The update rule and full table appear in S1 Appendix.

Third, policy transfer is evaluated across three multi-type environments: independent negative-binomial margins, a shared-gamma negative-binomial model and the finite-memory reinforced model. Each environment calibrates a can-order policy on a common grid; every calibrated policy is then scored in every test environment. This 3×3 design distinguishes marginal over-dispersion from dependence that affects coordination.

Fourth, the assumed design ratio *q*/*h* and the true evaluation ratio are crossed over {2,5,10,20}. The resulting regret matrix shows whether model choice remains important when economic inputs are misspecified.

### Simulation size, computation and reproducibility

All simulations use master random seed 20260531 and deterministic sub-seeds. Classical moment validation uses 12,000 explicit urn paths, and scalar demand experiments use 10,000–150,000 draws depending on the calculation. Monte Carlo confidence intervals are based on paired replications or rolling origins, as stated in each table. The design follows standard Monte Carlo and simulation-experiment practice [42–44], while the transparent policy grids provide a reproducible form of simulation optimization [45]. All analyses were rerun from a clean working directory. Source code, fixed aggregate data, environment versions, generated tables and an MIT license are supplied in S1 Code. No proprietary solver or external computing service is required.

## Results

### Count dispersion in two public datasets

RAND-HIE visit counts were over-dispersed in every health-status group (Table 1). The variance-to-mean ratio ranged from 6.43 in the excellent-health group to 9.66 in the poor-health group. About one third of respondents in the first three groups reported no visit. Their 95th percentiles were 9, 10 and 13 visits; the smaller poor-health group had a 95th percentile of 21. Maxima across the four groups ranged from 69 to 77 visits.

**Table 1:** Descriptive count summaries for the two public datasets. RAND-HIE rows are person-year observations; NYC 311 rows are daily category counts from 2022–2024.

Series	Observations	Mean	Variance	Variance/mean	Maximum
RAND: excellent health	11,019	2.634	16.941	6.43	74
RAND: good health	7,309	2.902	20.451	7.05	77
RAND: fair health	1,560	3.692	33.617	9.10	69
RAND: poor health	302	5.795	55.958	9.66	72
NYC: blocked driveway	1,096	449.356	2,530.102	5.63	655
NYC: illegal parking	1,096	1,266.861	40,027.559	31.60	1,773
NYC: residential noise	1,096	962.407	407,694.181	423.62	6,559
NYC: street condition	1,096	195.599	6,540.167	33.44	467
NYC: water system	1,096	166.598	14,086.137	84.55	966

The NYC series supplied a different scale and temporal structure. Daily category means ranged from 166.6 to 1,266.9, and every raw variance-to-mean ratio exceeded five. Residential noise was especially episodic, with a maximum daily count of 6,559 and a raw ratio of 423.6. These raw summaries contain seasonality and trend as well as residual variation, which is why the rolling analysis first estimates a calendar mean and calibrates the candidate residual generators on the preceding year.

**Fig 2:**
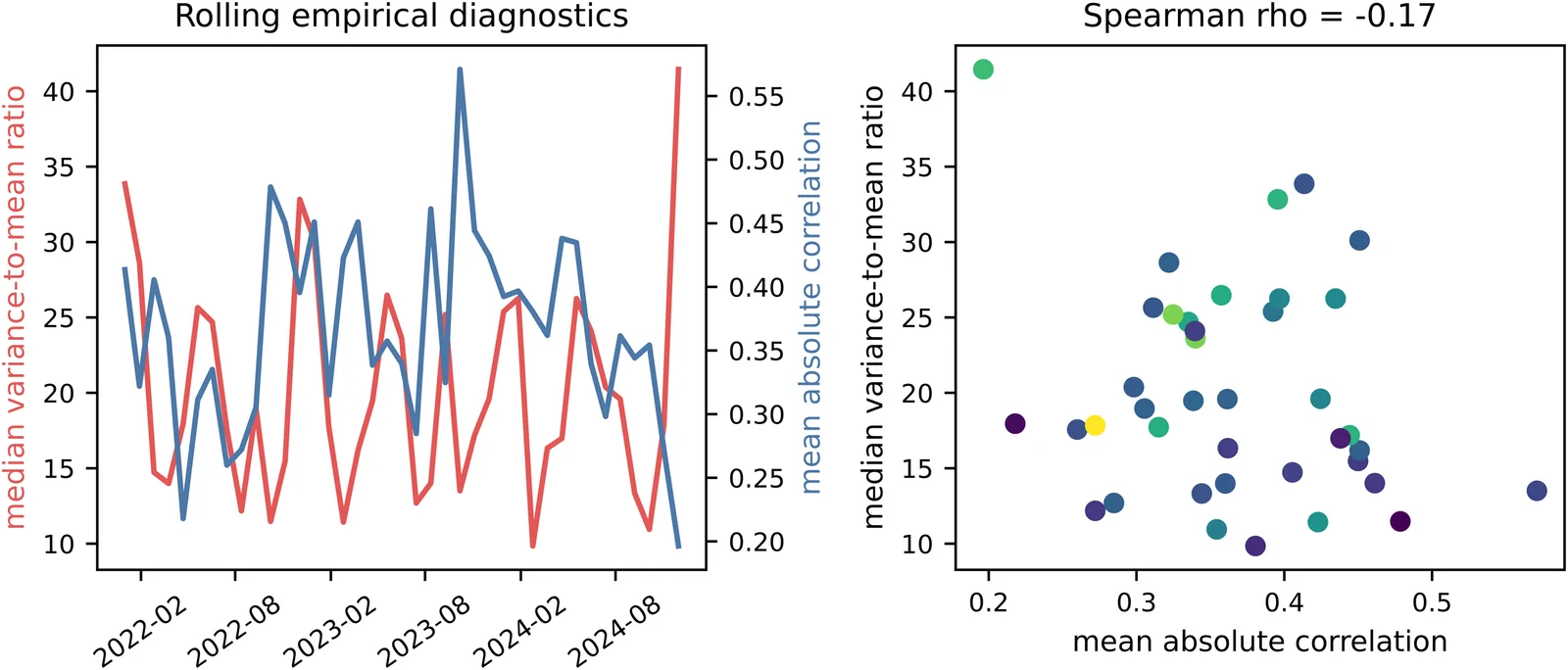
Rolling dispersion and correlation in NYC 311 counts. The left panel reports 28-day median variance-to-mean ratios and mean absolute cross-category correlations. The right panel shows that windows with greater dispersion do not systematically have greater pairwise correlation; point color represents the median 95th-percentile-to-mean ratio.

The 28-day NYC windows also illustrate why correlation alone is an incomplete diagnostic (Fig 2). Windows in the upper quartile of median dispersion had an average variance-to-mean ratio of 29.69 and a median 95th-percentile-to-mean ratio of 1.44. Their mean absolute cross-category correlation was 0.37, slightly below the 0.39 observed in the lower-dispersion quartile. Across all windows, the Spearman association between dispersion and absolute correlation was −0.175 (*p* = 0.287). This descriptive result shows that large changes in operational dispersion need not be accompanied by larger pairwise correlation in a real multi-type count panel.

### Cross-sectional held-out decisions in RAND-HIE

The RAND-HIE splits provide a direct held-out decision check (Table 2). At *q*/*h* = 5, differences were modest: the reinforced decision reduced cost by 1.11% and increased fill rate by about eight percentage points relative to Poisson. The gap widened as missed visits became more costly. At *q*/*h* = 20, Poisson had a held-out cost of 17.379 and fill rate of 76.48%; the reinforced decision had cost 14.757 and fill rate 91.14%, a paired cost improvement of 15.01%. Negative-binomial and empirical decisions performed similarly or better in this scalar setting. That agreement is expected because marginal dispersion is the principal feature affecting this scalar action.

**Table 2:** Cross-sectional held-out decision validation on RAND-HIE counts. Results average 200 stratified 50/50 splits. The improvement is the paired cost reduction relative to independent Poisson; regret is measured against the held-out oracle.

*q*/*h*	Decision model	Held-out cost	Fill (%)	Improvement (%)	Regret (%)
5	Independent Poisson	7.269	66.50	0.00	1.70
5	Negative binomial	7.181	74.25	1.20	0.48
5	Reinforced count	7.187	74.48	1.11	0.56
5	Empirical	7.152	72.23	1.60	0.07
10	Independent Poisson	11.217	71.67	0.00	8.90
10	Negative binomial	10.462	85.25	6.72	1.58
10	Reinforced count	10.492	85.54	6.45	1.87
10	Empirical	10.326	82.31	7.94	0.25
20	Independent Poisson	17.379	76.48	0.00	21.26
20	Negative binomial	14.650	90.78	15.64	2.25
20	Reinforced count	14.757	91.14	15.01	3.01
20	Empirical	14.370	88.20	17.27	0.28

### External rolling-origin validation

**Fig 3:**
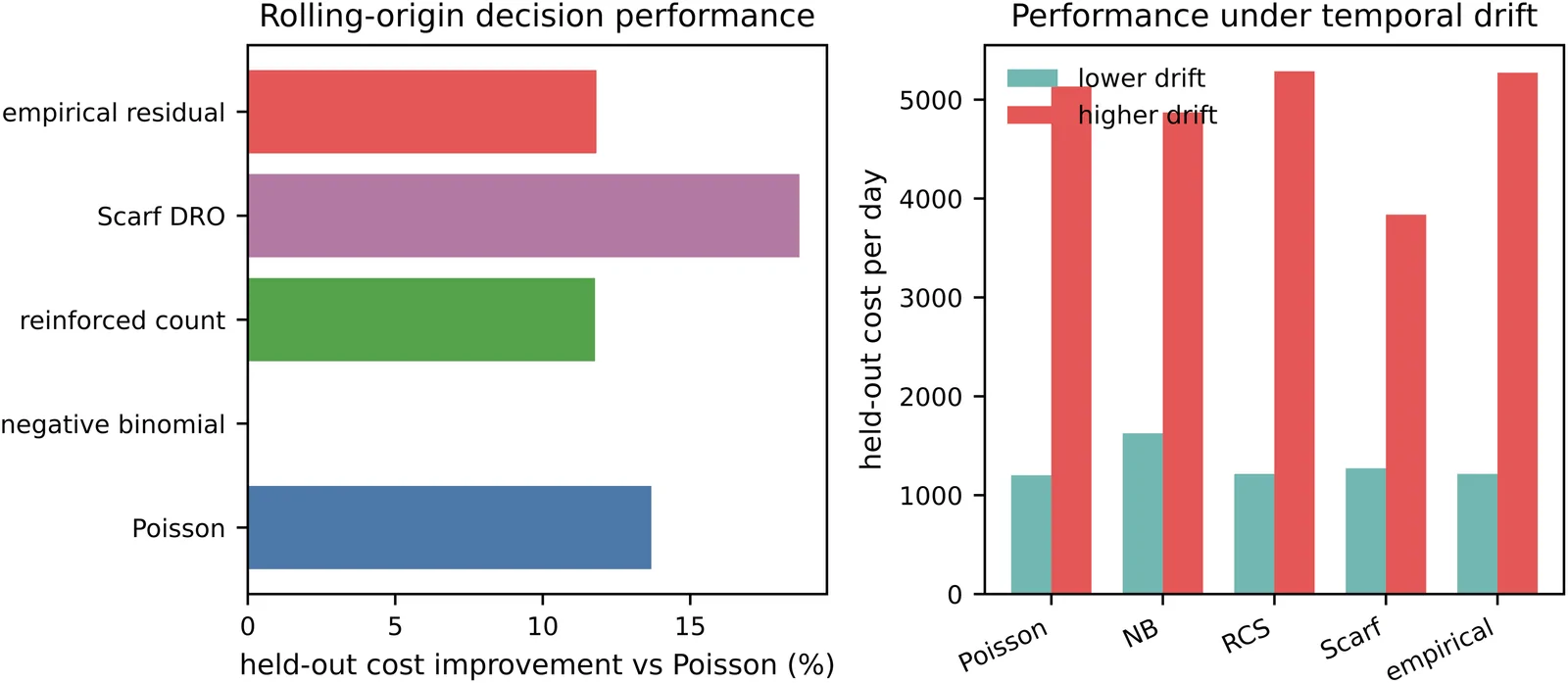
NYC 311 rolling-origin decision validation at *q*/*h* = 10. The left panel reports mean paired held-out cost improvement relative to independent Poisson across 26 origins. The right panel separates origins below and above the median temporal-drift index.

The NYC analysis evaluates decisions in future, time-ordered windows (Table 3 and Fig 3). The 26 training estimates of θ had median 199.0 and interquartile range 123.7–236.6, corresponding to weak composition reinforcement (ρ≈0.005). Even weak share variation can affect high-volume counts because a small proportional change moves many events.

**Table 3:** External rolling-origin validation on five NYC 311 categories. Cost and fill are means across 26 test origins. Improvement is first calculated within each origin relative to Poisson and then averaged, giving each origin equal weight; Cost/day is instead the mean of absolute origin costs. Origins with different cost scales can therefore yield a positive mean paired improvement even when the displayed overall mean cost is higher.

*q*/*h*	Decision model	Cost/day	Fill (%)	Improvement, 95% CI (%)
5	Independent Poisson	2,234.14	92.78	0.00
5	Negative binomial	2,454.24	95.50	0.68 (−8.24, 9.59)
5	Reinforced count	2,039.39	95.73	7.70 (2.38, 13.02)
5	Empirical residual	2,281.37	94.76	5.71 (−0.24, 11.65)
5	Scarf moment-DRO	2,446.22	95.33	1.00 (−7.51, 9.51)
10	Independent Poisson	3,247.60	93.26	0.00
10	Negative binomial	3,250.18	96.43	11.77 (0.40, 23.14)
10	Reinforced count	2,553.94	96.82	18.70 (12.36, 25.04)
10	Empirical residual	3,166.70	95.78	13.68 (3.98, 23.38)
10	Scarf moment-DRO	3,243.65	96.52	11.82 (0.30, 23.34)

At *q*/*h* = 5, RCS improved held-out cost by a paired mean of 7.70% (95% CI, 2.38%–13.02%) and increased fill from 92.78% to 95.73%. At *q*/*h* = 10, the improvement was 18.70% (95% CI, 12.36%–25.04%) and fill increased from 93.26% to 96.82%. The negative-binomial, empirical and Scarf decisions also improved the paired mean at *q*/*h* = 10, but with wider intervals. Percentages were calculated within each origin before averaging, whereas Cost/day is the mean of absolute origin costs. Origins with larger absolute costs therefore contribute more to the latter comparison, which explains how a positive mean paired percentage can coexist with a higher displayed mean cost. RCS retained its advantage in both halves of the drift distribution: its mean cost per day was 1,271 versus 1,625 for Poisson in lower-drift origins, and 3,836 versus 4,870 in higher-drift origins.

### Multi-type model transfer

**Fig 4:**
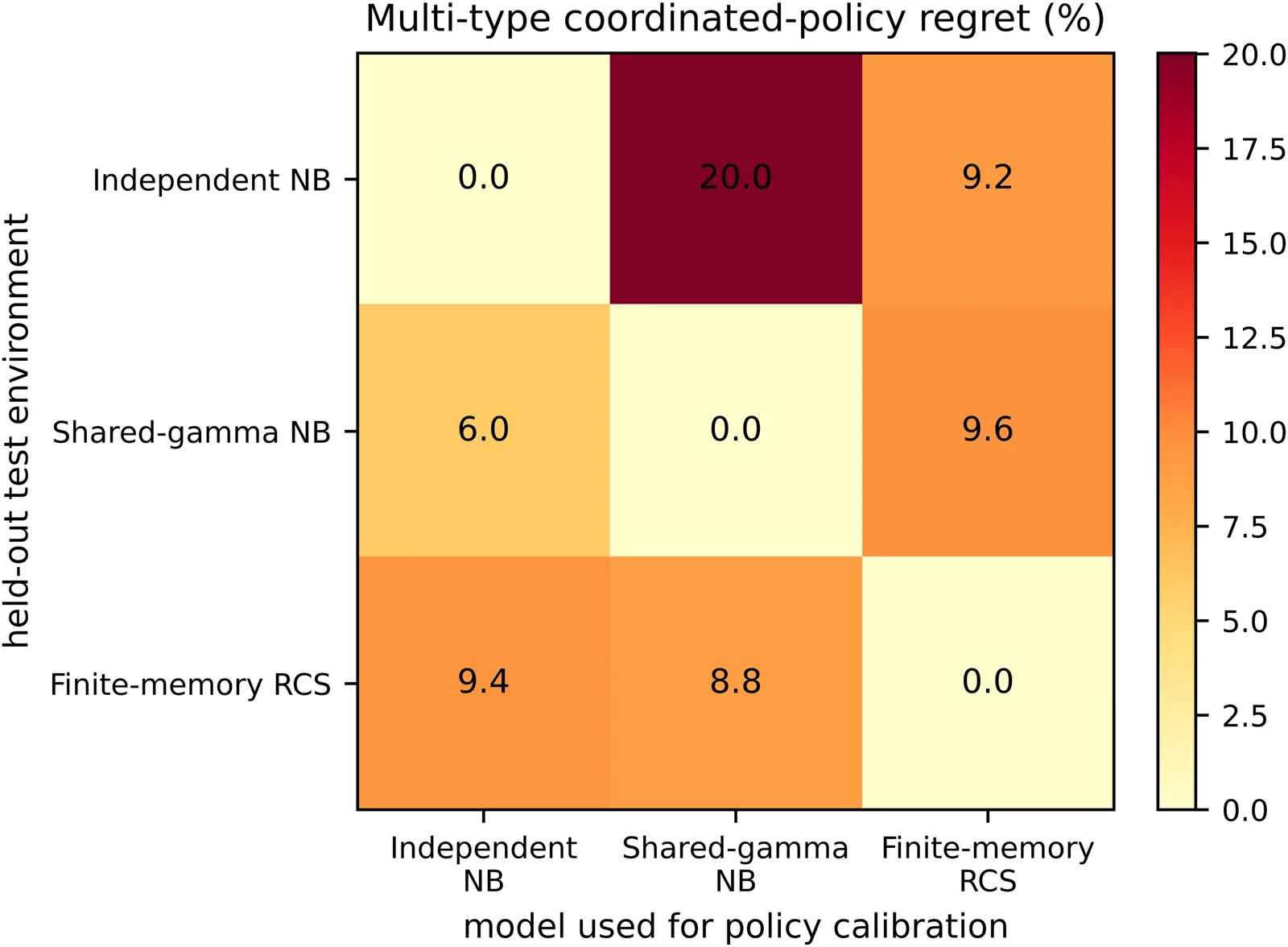
Multi-type coordinated-policy regret. Columns identify the model used to calibrate the can-order policy, and rows identify the held-out test environment. Diagonal cells are zero by construction because each row is compared with the best of the three calibrated policies in that environment.

The coordinated-policy experiment clarifies what RCS contributes beyond a scalar negative-binomial correction (Table 4 and Fig 4). Each model performed best when policy calibration and test environment matched. The independent negative-binomial policy incurred 5.95% regret under shared-gamma demand and 9.43% under finite-memory reinforced demand. The shared-gamma policy incurred 20.02% regret under independent negative-binomial demand and 8.85% under reinforced demand. The reinforced policy incurred 9.18%–9.58% regret in the two negative-binomial environments.

**Table 4:** Transfer of calibrated can-order policies across multi-type count environments. Regret is relative to the lowest-cost candidate policy within each test row.

Test environment	Calibration model	Policy	Cost	Regret (%)	Fill (%)
Independent NB	Independent NB	(4,10,14)	46.50	0.00	96.89
Independent NB	Shared-gamma NB	(8,14,18)	55.82	20.02	99.46
Independent NB	Finite-memory RCS	(6,6,14)	50.77	9.18	96.98
Shared-gamma NB	Independent NB	(4,10,14)	86.67	5.95	80.20
Shared-gamma NB	Shared-gamma NB	(8,14,18)	81.80	0.00	88.20
Shared-gamma NB	Finite-memory RCS	(6,6,14)	89.63	9.58	80.04
Finite-memory RCS	Independent NB	(4,10,14)	67.74	9.43	90.89
Finite-memory RCS	Shared-gamma NB	(8,14,18)	67.38	8.85	97.17
Finite-memory RCS	Finite-memory RCS	(6,6,14)	61.90	0.00	93.08

Matching marginal variance alone did not identify a coordinated policy: each model was cheapest in its matching environment, and mismatch changed the selected can-order parameters, setup frequency and fill rate.

### Joint matrix sensitivity

**Fig 5:**
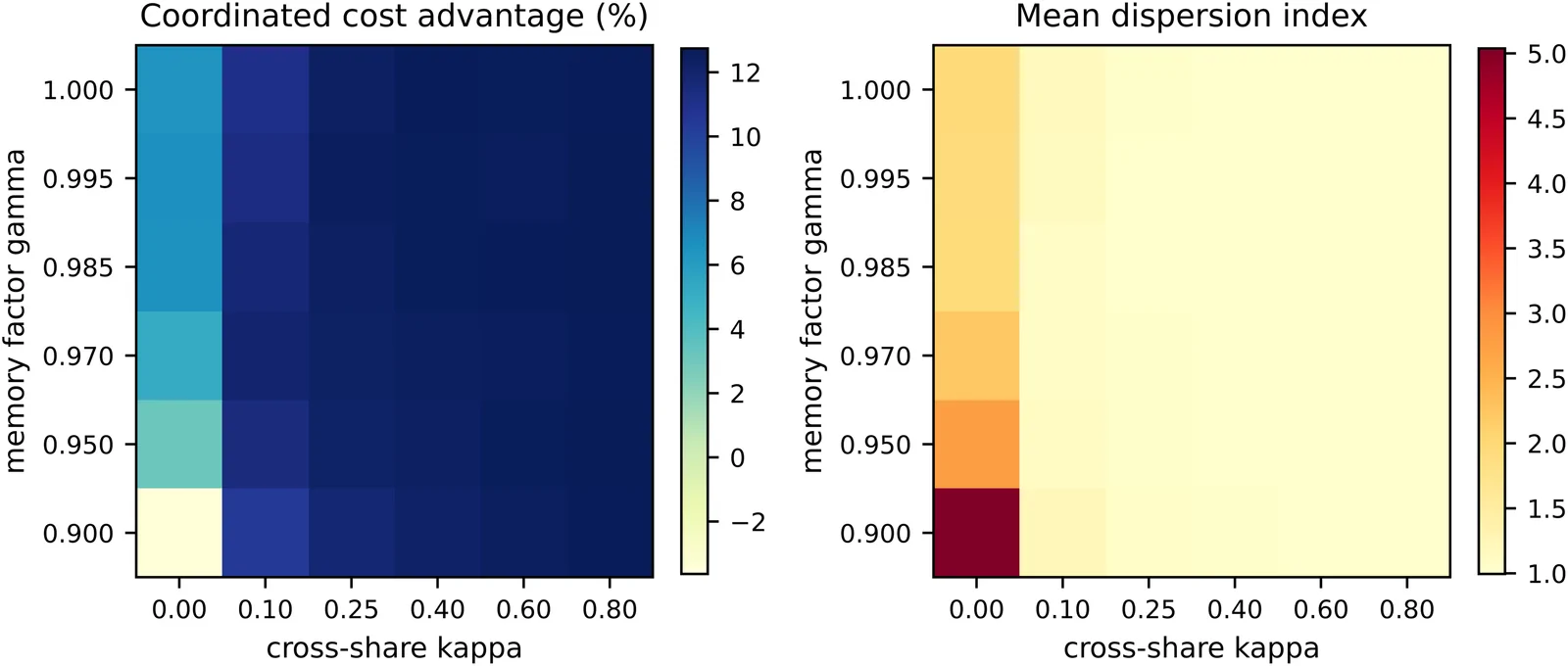
Joint sensitivity to cross-share κ and memory γ. Panels report dispersion, lag-one autocorrelation and coordinated can-order cost advantage over the complete 6×6 grid. The response to κ is not equivalent to a simple weakening of reinforcement.

Fig 5 replaces one-parameter-at-a-time checks with the full κ×γ grid. With no cross-share (κ=0), shorter memory produced substantial marginal dispersion and autocorrelation; at γ=0.90, the mean dispersion index was 5.04 and lag-one autocorrelation was 0.81. In that corner, the coordinated rule cost 3.63% more than separate replenishment because strong within-type persistence did not create a setup-sharing opportunity.

Once κ≥0.10, dispersion moved closer to one, type-specific persistence weakened and the coordinated cost advantage was generally 10.4%–12.7% across γ. Fill rates were already high (mostly 0.993–0.998), so the advantage came mainly from sharing the fixed setup charge, with little service-level change. The full numerical grid, including confidence intervals, appears in S1 Appendix.

### Concentration learning and break-even stress tests

**Fig 6:**
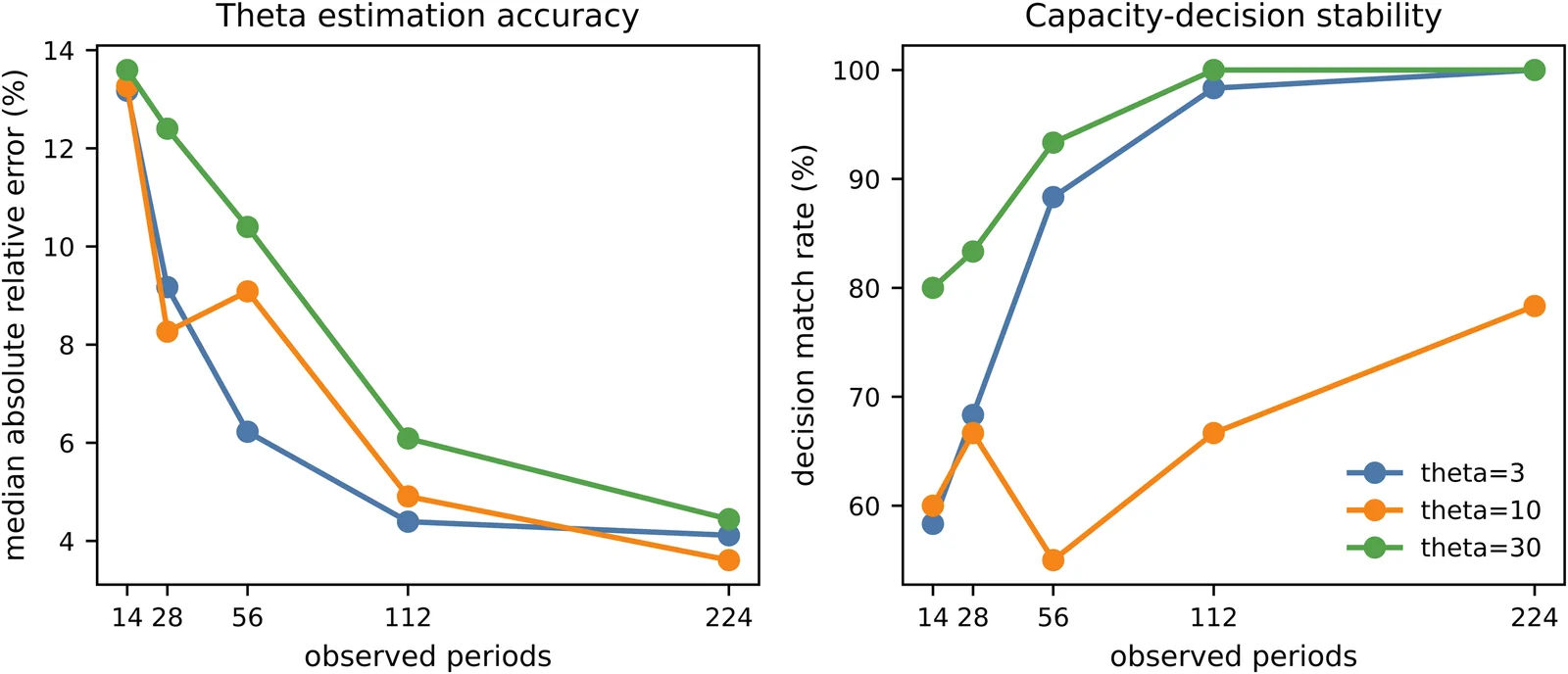
Concentration estimation and decision stability. The left panel shows median absolute relative error in θ. The right panel shows the percentage of simulated samples selecting exactly the same capacity as the true-θ model. Across all conditions, a separate within-one-capacity-unit measure was at least 96.7%; exact recovery of θ requires substantially more data. Each point contains 60 replications.

The simulation learning curves in Fig 6 quantify the data requirement for estimating θ. With 14 periods, the median absolute relative error was 13.2%–13.6% across the three true values. With 224 periods it fell to 3.6%–4.4%. More importantly for the intended use, at least 96.7% of decisions were within one capacity unit of the true-θ decision even at 14 periods, and the rate was 100% in all but one design cell. This result supports operational decision stability; precise recovery of θ requires substantially more data. Exact equality can be non-monotone because a small parameter error may cross an integer quantile boundary, making the within-one-unit result the more useful operational measure.

When no count sample is available, the break-even calculation gives an interpretable stress range. For Λ=12 and *q*/*h* = 10, the independent capacity was 8 and the reinforced decision first changed at θ≈14.63 (ρ≈0.064). At Λ=20, the corresponding threshold was θ≈36.75. At Λ=40, a one-unit change appeared even near the upper grid boundary θ=500 (ρ≈0.002), showing that weak proportional heterogeneity can matter when volume is large. The full 4×4 break-even table is included in S1 Appendix.

### Cost-parameter uncertainty

**Fig 7:**
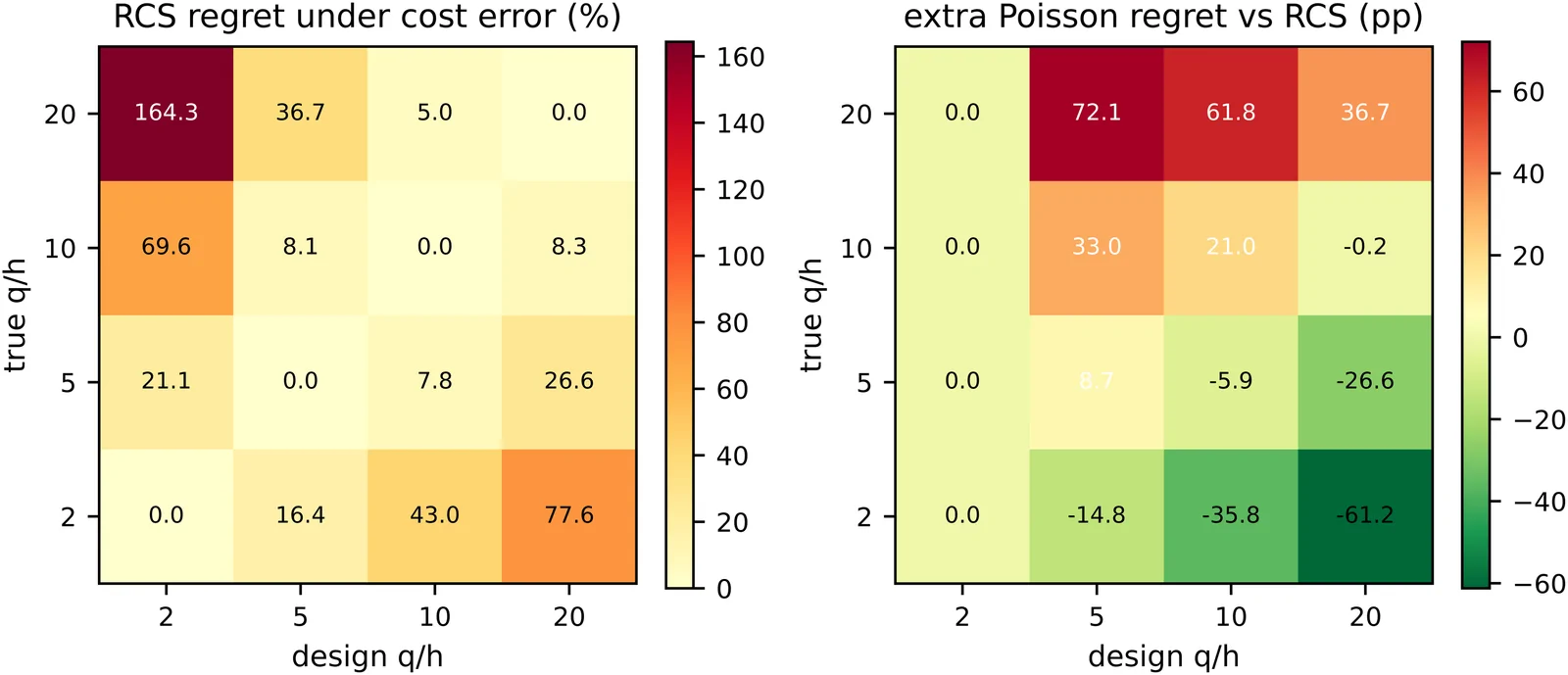
Decision regret under cost-ratio error. The left panel gives reinforced-count regret for each combination of design and true *q*/*h*. The right panel subtracts reinforced regret from Poisson regret; positive cells favor RCS and negative cells favor Poisson.

Fig 7 shows that uncertainty about *q*/*h* is a separate source of decision risk. When the design and true ratios both equaled 10, the reinforced decision matched the oracle and the Poisson decision had 20.99% regret. When the design ratio remained 10 but the true ratio was 20, regret was 5.02% for RCS and 66.87% for Poisson. In contrast, if the true ratio was only 2, the same RCS decision over-provided capacity and incurred 43.05% regret, compared with 7.25% for Poisson. Reinforcement-aware calibration can therefore be economically misaligned when *q*/*h* is overstated. Analysts should vary model structure and cost inputs jointly.

### Computational overhead

The additional analyses completed in about 40 seconds on the reported Windows workstation (Table 5). The external rolling validation, which repeatedly fits five calendar models and simulates five decision generators, required 9.4 seconds. The full matrix grid required 15.8 seconds, and multi-type policy transfer required 10.7 seconds. These times are small relative to weekly or monthly planning cycles. For high-frequency real-time control or very large policy spaces, a precomputed stress grid or an analytic quantile approximation would be more appropriate than rerunning the full simulation at each event.

**Table 5:** Observed runtime for the additional revision analyses. Times are wall-clock seconds under Python 3.8.5, NumPy 1.24.4, pandas 2.0.3 and statsmodels 0.14.1.

Analysis	Runtime (s)
NYC rolling-origin validation	9.43
Joint κ×γ sensitivity	15.78
Multi-type calibration transfer	10.73
Cost-ratio uncertainty	1.30
θ learning curve	2.65
Total	39.89

## Discussion

### Principal findings

An independence-calibrated decision was fragile under unresolved count heterogeneity in both a cross-sectional healthcare sample and a time-ordered municipal service panel. The NYC result is especially informative because each future window was separated from its one-year calibration window and the origins varied substantially in drift.

No candidate model was uniformly best. Negative-binomial and empirical decisions were competitive in scalar settings, and each multi-type calibration model was best in its matching environment. RCS broadens the set of plausible environments used to test a consequential decision.

### Relation to distributionally robust decisions

RCS and DRO answer related but distinct questions. The Scarf decision protects against every distribution with a specified mean and variance and therefore offers a formal worst-case guarantee for a scalar loss. The comparison provides methodological transparency: a classical moment-robust rule already captured part of the cost reduction, while RCS also represented multivariate composition dynamics. RCS specifies a mechanism for composition uncertainty, allowing simulated type competition, persistence, finite memory and policy interaction. Its simulated demand paths can be inserted into an existing operational model without solving a new robust optimization problem.

Moment-DRO is less dependent on a particular stochastic mechanism, while RCS can be misleading when reinforced composition is a poor stress direction. Wasserstein and other data-driven ambiguity sets provide richer alternatives when sufficient observations and an appropriate optimization model are available [8,20]. In practice, RCS should be used alongside robust and mixed-count benchmarks. Agreement across them is reassuring; disagreement identifies a decision that requires more data or domain review.

### Data requirements and return on computation

The minimum viable input is a defensible exposure-adjusted mean vector, clearly defined event types and an estimated range for *q*/*h*. With only these inputs, the method can report a θ break-even curve, but it cannot estimate reinforcement. Estimation additionally requires repeated multi-type counts measured on a consistent time scale. Calendar, exposure and known policy effects should be removed from the mean before residual reinforcement is interpreted. Time stamps are needed when the intended decision will face drift, and jointly observed type counts are needed when coordination is at issue.

There is no universal sample-size cutoff. The learning experiment suggests that 14–28 repeated periods can support a coarse decision stress test in the simulated designs; precise parameter inference requires longer samples. More observations do not correct biased measurement, omitted exposure or a policy change that altered how requests were recorded. A useful deployment rule is to proceed only when (i) the independence action changes over a plausible θ range, (ii) the implied cost difference exceeds the organization’s tolerance, and (iii) the result is stable to cost and mean perturbations. If the action never changes, the simulation has little return. If it changes sharply, the diagnostic has identified where additional data collection is valuable.

For periodic planning, the observed computational burden is modest. The code uses exhaustive integer grids and standard simulation on general-purpose hardware. Real-time applications with many items would benefit from screening items by volume, over-dispersion and cost asymmetry, then running RCS only for the subset near a decision boundary.

### Extensions to forecasting and deep learning

RCS is compatible with more flexible mean forecasts. A deep or machine-learning model can estimate $\mu_{t}$ from calendar, clinical, customer or equipment features; reinforced simulation can then stress-test the residual composition around that forecast. This separates nonlinear prediction from the evaluation of tail-sensitive decisions. Deep learning has been used to combine heterogeneous healthcare signals for detection and classification [46]; analogous representations could enter the mean or state model for service demand. Such combinations require temporally separated validation and calibration checks, with operational benefit evaluated separately from predictive accuracy.

### Practical implications

Applying the workflow to outpatient, call-center or spare-parts settings would require domain-specific definitions of exposure, event types and costs, followed by validation on the target system. Event types might then be specialties, channels, request classes, part families or failure modes. The analyst first fits known exposure and seasonality, then checks residual dispersion and evaluates the current staffing or reorder rule under Poisson, mixed-count, reinforced and robust alternatives. The output should identify the selected action, the cost components, the fill or service level and the range of parameter values that changes the recommendation.

The matrix experiment adds a specific caution for coordinated systems. A high fill rate can coexist with valuable coordination: in our high-service design, most of the advantage came from fixed-setup sharing. Conversely, strong within-type persistence with no cross-share did not favor the can-order policy. Practitioners should therefore report holding, shortage and setup components instead of interpreting a single total-cost percentage as a service improvement.

### Limitations

Several limitations bound the conclusions. RAND-HIE is cross-sectional and historical, so it cannot test temporal transfer. NYC 311 supplies temporal evidence from an administrative request system whose operating conditions differ from hospital or inventory records, and the public source may revise historical entries. The included fixed aggregate snapshot preserves the analyzed version. Neither dataset supplies a verified economic *q*/*h*; the reported ratios are decision scenarios. The cost-uncertainty experiment shows that this assumption can dominate model choice.

The Scarf comparator represents classical moment-DRO, not the full range of modern ambiguity sets. The finite-memory matrix urn remains an exploratory simulator without an exchangeable limit theory. The empirical dispersion-correlation result illustrates diagnostic decoupling but does not prove that either public dataset follows an urn. Finally, the candidate mean model for NYC is intentionally simple. A stronger operational study should combine domain-specific forecasting, richer exposure data, recorded staffing or inventory actions, and prospective evaluation.

## Conclusions

Reinforced-count simulation provides a reproducible way to ask whether an independence-based capacity or inventory decision is sensitive to unresolved composition heterogeneity. Across RAND-HIE held-out counts and 26 NYC 311 rolling origins, over-dispersion-aware decisions often reduced shortage-sensitive cost and increased fill. Comparisons with negative-binomial, empirical and moment-robust decisions attribute part of this gain to shared over-dispersion correction, while multi-type transfer demonstrates that dependence structure can matter when decisions are coordinated.

RCS is most informative when demand volume or lost-event cost is high, the decision changes over a plausible reinforcement range and the available data are insufficient to settle the dependence model. The calibration curves, cost perturbations, runtime results and open code make the diagnostic reproducible.

## Supporting information

S1 Appendix Mathematical and computational details. Classical urn derivations, model definitions, complete simulation settings, supplementary tables, and additional validation results.
(PDF)

S1 Code Reproducibility package. A ZIP archive containing documented Python source, exact package versions, the fixed NYC aggregate, the public-data download query, generated numerical tables, expected-output checksums and an open-source license. Running the two analysis scripts from the package root regenerates every numerical result and all figures.
(ZIP)

## References

[1] Scarf H. Bayes solutions of the statistical inventory problem. Ann Math Stat. 1959;30(2):490–508. doi: 10.1214/aoms/1177706264

[2] Azoury KS. Bayes solution to dynamic inventory models under unknown demand distribution. Manag Sci. 1985;31(9):1150–60. doi: 10.1287/mnsc.31.9.1150

[3] Eppen GD. Effects of centralization on expected costs in a multi-location newsboy problem. Manag Sci. 1979;25(5):498–501. doi: 10.1287/mnsc.25.5.498

[4] Liyanage LH, Shanthikumar JG. A practical inventory control policy using operational statistics. Oper Res Lett. 2005;33(4):341–8. doi: 10.1016/j.orl.2004.08.003

[5] Levi R, Perakis G, Uichanco J. The data-driven newsvendor problem: new bounds and insights. Oper Res. 2015;63(6):1294–306. doi: 10.1287/opre.2015.1422

[6] Ban GY, Rudin C. The big data newsvendor: practical insights from machine learning. Oper Res. 2019;67(1):90–108. doi: 10.1287/opre.2018.1757

[7] Besbes O, Mouchtaki O. How big should your data really be? Data-driven newsvendor: Learning one sample at a time. Manag Sci. 2023;69(10):5848–65. doi: 10.1287/mnsc.2023.4725

[8] Xu L, Zheng Y, Jiang L. A robust data-driven approach for the newsvendor problem with nonparametric information. Manuf Serv Oper Manag. 2022;24(1):504–23. doi: 10.1287/msom.2020.0961

[9] Silver EA. A control system for coordinated inventory replenishment. Int J Prod Res. 1974;12(6):647–71. doi: 10.1080/00207547408919583

[10] Goyal SK, Satir AT. Joint replenishment inventory control: deterministic and stochastic models. Eur J Oper Res. 1989;38(1):2–13. doi: 10.1016/0377-2217(89)90463-3

[11] Khouja M, Goyal S. A review of the joint replenishment problem literature: 1989–2005. Eur J Oper Res. 2008;186(1):1–16. doi: 10.1016/j.ejor.2007.03.007

[12] Taylor JW. A comparison of univariate time series methods for forecasting intraday arrivals at a call center. Manag Sci. 2008;54(2):253–65. doi: 10.1287/mnsc.1070.0786

[13] Ibrahim R, Ye H, L’Ecuyer P, Shen H. Modeling and forecasting call center arrivals: a literature survey and a case study. Int J Forecast. 2016;32(3):865–74. doi: 10.1016/j.ijforecast.2015.11.012

[14] Aksin Z, Armony M, Mehrotra V. The modern call center: a multi-disciplinary perspective on operations management research. Prod Oper Manag. 2007;16(6):665–88. doi: 10.1111/j.1937-5956.2007.tb00288.x

[15] Channouf N, L’Ecuyer P, Ingolfsson A, Avramidis AN. The application of forecasting techniques to modeling emergency medical system calls in Calgary, Alberta. Health Care Manag Sci. 2007;10(1):25–45. doi: 10.1007/s10729-006-9006-3 17323653

[16] Reboredo JC, Barba-Queiruga JR, Ojea-Ferreiro J, Reyes-Santias F. Forecasting emergency department arrivals using INGARCH models. Health Econ Rev. 2023;13(1):51. doi: 10.1186/s13561-023-00456-5 37897674 PMC10612291

[17] Bertsimas D, Kallus N. From predictive to prescriptive analytics. Manag Sci. 2020;66(3):1025–44. doi: 10.1287/mnsc.2018.3253

[18] Snyder LV, Atan Z, Peng P, Rong Y, Schmitt AJ, Sinsoysal B. OR/MS models for supply chain disruptions: a review. IIE Trans. 2015;48(2):89–109. doi: 10.1080/0740817x.2015.1067735

[19] Scarf H. A min-max solution of an inventory problem. In: Arrow KJ, Karlin S, Scarf H, editors. Studies in the mathematical theory of inventory and production. Stanford: Stanford University Press; 1958. p. 201–9.

[20] Gao R, Kleywegt AJ. Distributionally robust stochastic optimization with Wasserstein distance. Math Oper Res. 2023;48(2):603–55. doi: 10.1287/moor.2022.1275

[21] Kourentzes N. On intermittent demand model optimisation and selection. Int J Prod Econ. 2014;156:180–90. doi: 10.1016/j.ijpe.2014.06.007

[22] Winkelmann R. Econometric analysis of count data. 5th ed. Berlin: Springer; 2008.

[23] Cameron AC, Trivedi PK. Regression analysis of count data. 2nd ed. Cambridge: Cambridge University Press; 2013. doi: 10.1017/CBO9781139013567

[24] Lord D, Mannering F. The statistical analysis of crash-frequency data: a review and assessment of methodological alternatives. Transp Res A: Policy Pract. 2010;44(5):291–305. doi: 10.1016/j.tra.2010.02.001

[25] Sellers KF, Shmueli G. A flexible regression model for count data. Ann Appl Stat. 2010;4(2):943–61. doi: 10.1214/09-AOAS306

[26] Nelsen RB. An introduction to copulas. 2nd ed. New York: Springer; 2006. doi: 10.1007/0-387-28678-0

[27] Petropoulos F, Apiletti D, Assimakopoulos V. Forecasting: theory and practice. Int J Forecast. 2022;38(3):705–871. doi: 10.1016/j.ijforecast.2021.11.001

[28] Eggenberger F, Polya G. Uber die Statistik verketteter Vorgange. Z Angew Math Mech. 1923;3(4):279–89. doi: 10.1002/zamm.19230030407

[29] de Finetti B. La prevision: ses lois logiques, ses sources subjectives. Ann Inst Henri Poincare. 1937;7(1):1–68.

[30] Blackwell D, MacQueen JB. Ferguson distributions via Polya urn schemes. Ann Stat. 1973;1(2):353–5. doi: 10.1214/aos/1176342372

[31] Aldous DJ. Exchangeability and related topics. In: Ecole d’Ete de Probabilites de Saint-Flour XIII–1983. Lecture Notes in Mathematics 1117. Berlin: Springer; 1985. p. 1–198.

[32] Hisakado M, Hattori K, Mori S. From the multiterm urn model to the self-exciting negative binomial distribution and Hawkes processes. Phys Rev E. 2022;106(3–1):034106. doi: 10.1103/PhysRevE.106.034106 36266900

[33] Johnson NL, Kotz S. Urn models and their application: an approach to modern discrete probability theory. New York: Wiley; 1977.

[34] Mahmoud HM. Polya urn models. Boca Raton: Chapman & Hall/CRC; 2008. doi: 10.1201/9781420059847

[35] Pemantle R. A survey of random processes with reinforcement. Probab Surv. 2007;4:1–79. doi: 10.1214/07-PS094

[36] Janson S. Functional limit theorems for multitype branching processes and generalized Polya urns. Stoch Process Their Appl. 2004;110(2):177–245. doi: 10.1016/j.spa.2003.12.002

[37] Mosimann JE. On the compound multinomial distribution, the multivariate β- distribution, and correlations among proportions. Biometrika. 1962;49(1/2):65. doi: 10.2307/2333468

[38] Seabold S, Perktold J. Statsmodels: econometric and statistical modeling with Python. Proceedings of the 9th Python in Science Conference; 2010. 10.25080/Majora-92bf1922-011

[39] Cameron AC, Trivedi PK. Microeconometrics: methods and applications. Cambridge: Cambridge University Press; 2005.

[40] RAND Corporation. RAND Health Insurance Experiment [Internet]. Santa Monica: RAND Corporation; [cited 2026 Jun 11]. Available from: https://www.rand.org/health-care/projects/hie.html

[41] New York City Open Data. 311 Service Requests from 2020 to Present [Internet]. New York: City of New York; [cited 2026 Aug 20]. Available from: https://data.cityofnewyork.us/d/erm2-nwe9

[42] Law AM. Simulation modeling and analysis. 5th ed. New York: McGraw-Hill; 2015.

[43] Glasserman P. Monte Carlo methods in financial engineering. New York: Springer; 2004. doi: 10.1007/978-0-387-21617-1

[44] Kleijnen JPC. Design and analysis of simulation experiments. 2nd ed. New York: Springer; 2015. doi: 10.1007/978-3-319-18087-8

[45] Amaran S, Sahinidis NV, Sharda B, Bury SJ. Simulation optimization: a review of algorithms and applications. 4OR. 2016;14(4):301–33. doi: 10.1007/s10288-015-0292-x

[46] Goswami SS, Prasad A. Comparison among deep learning approaches and biomarkers in early detection of Parkinson’s disease. J Comput Cogn Eng. 2025;4(2):132–41. doi: 10.47852/bonviewJCCE42023040

