# Supplementary material for: Reinforced-count simulation for decision calibration under over-dispersed multi-type service demand
Source: PLoS One. 2026 Sep 18;21(9):e0358767. doi: 10.1371/journal.pone.0358767 (PMC13588529; doi:10.1371/journal.pone.0358767)
Supplement: Supplementary file 1 [file pone.0358767.s001.pdf]

S1 Appendix. Mathematical and computational details

Reinforced-count simulation for decision calibration under over-dispersed multi-type service demand

Saisai Hou, Yunzhi Zhu, Sen Zhang, Ying Chen

A. Purpose and scope

This appendix records the definitions, derivations, parameter settings and supplementary results underlying the main manuscript. The classical balanced-urn identities are included for completeness and code verification; they are not presented as new theorems. The matrix-urn and finite-memory models are simulation devices used to explore policy sensitivity, not fitted stochastic laws for either public dataset.

B. Classical arrival-level urn

Consider K event types. The urn initially contains $a_i > 0$ units of type i, with total $a_0 = \sum_i a_i$ and baseline proportion $p_i = a_i/a_0$. At each arrival, a type is sampled in proportion to its current weight, the sampled unit is returned, and $\alpha > 0$ additional units of that type are added. The dimensionless concentration is

$$\theta = \frac{a_0}{\alpha}.$$

Small θ represents strong reinforcement; $\theta \to \infty$ approaches independent categorical sampling with fixed probabilities $\boldsymbol{p}$. If X_t is the type of arrival t, the cumulative type count after m arrivals is

$$D_i(m) = \sum_{t=1}^{m} \mathbf{1}\{X_t = i\}.$$

The balanced urn admits a de Finetti representation

$$\boldsymbol{V} \sim \text{Dirichlet}(\theta \boldsymbol{p}), \qquad \boldsymbol{D}(m) \mid \boldsymbol{V} \sim \text{Multinomial}(m, \boldsymbol{V}).$$

For the Dirichlet directing vector,

$$\begin{aligned} E(V_i) &= p_i, \\ \text{Var}(V_i) &= \frac{p_i(1-p_i)}{\theta+1}, \\ \text{Cov}(V_i, V_j) &= -\frac{p_i p_j}{\theta+1}, \qquad i \neq j. \end{aligned}$$

The law of total variance gives

$$\begin{aligned} \text{Var}\{D_i(m)\} &= E\{mV_i(1-V_i)\} + \text{Var}(mV_i) \\ &= p_i(1-p_i)\frac{m(m+\theta)}{\theta+1}. \end{aligned}$$

Similarly,

$$\text{Cov}\{D_i(m), D_j(m)\} = -p_i p_j \frac{m(m+\theta)}{\theta+1}.$$

The common multiplier cancels from the normalized correlation:

$$\mathrm{Corr}\{D_i(m), D_j(m)\} = -\sqrt{\frac{p_i p_j}{(1-p_i)(1-p_j)}}.$$

This invariance applies to cumulative counts under the balanced classical urn. It does not imply that empirical correlations are generally invariant to all forms of heterogeneity.

Table 1: Monte Carlo validation of the classical moments at $m = 220$. Each finite-θ condition uses 12,000 explicit urn paths.

θ	Empirical variance	Theory	Relative error (%)	Empirical corr.	Theory
1	3968.712	3889.600	2.03	-0.2572	-0.2500
2	2554.325	2604.800	1.94	-0.2499	-0.2500
5	1345.600	1320.000	1.94	-0.2440	-0.2500
∞	35.692	35.200	1.40	-0.2383	-0.2500

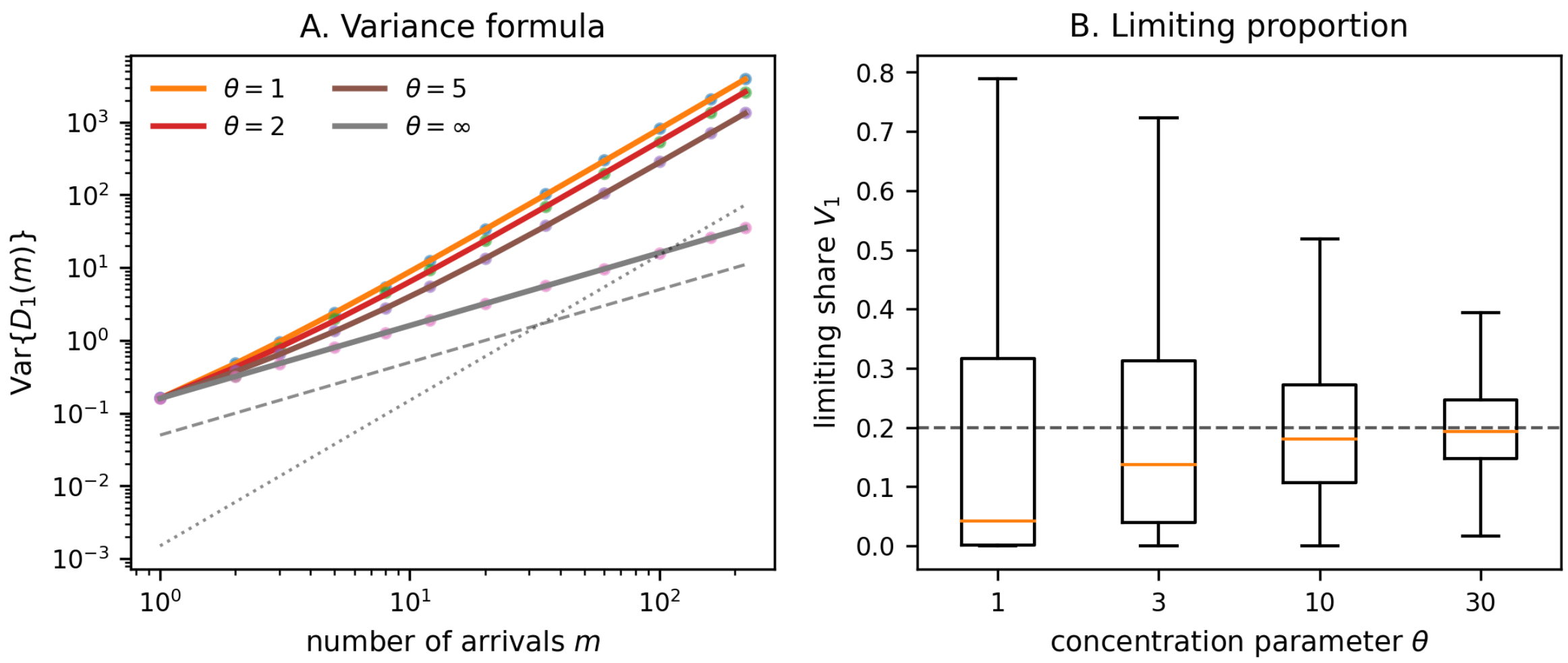

Figure 1: Classical urn diagnostics. The panels compare empirical and theoretical count moments and show limiting composition variability.

C. Period-level count generators

The period experiments separate expected total volume Λ_t from the baseline type shares $\boldsymbol{p}_t$. The independent model uses $D_{ti} \sim \mathrm{Poisson}(\Lambda_t p_{ti})$. The gamma-Poisson model draws an independent random rate for each type, yielding

$$E(D_{ti}) = \mu_{ti}, \qquad \mathrm{Var}(D_{ti}) = \mu_{ti} + \alpha_i \mu_{ti}^2.$$

The reinforced period generator draws $\boldsymbol{V}_t \sim \mathrm{Dirichlet}(\theta \boldsymbol{p}_t)$ and then conditionally independent Poisson counts with means $\Lambda_t V_{ti}$. Its unconditional moments are

$$\begin{aligned} E(D_{ti}) &= \Lambda_t p_{ti}, \\ \mathrm{Var}(D_{ti}) &= \Lambda_t p_{ti} + \Lambda_t^2 \frac{p_{ti}(1-p_{ti})}{\theta+1}, \\ \mathrm{Cov}(D_{ti}, D_{tj}) &= -\Lambda_t^2 \frac{p_{ti} p_{tj}}{\theta+1}. \end{aligned}$$

For scalar stock decisions, a negative-binomial predictive and a reinforced beta-Poisson predictive may select the same integer quantile when their marginal mean and dispersion are close. They remain different distributions, and the multivariate reinforced model also imposes composition competition.

Table 2: Supplementary scalar benchmark under $\Lambda = 12$, $\theta = 2$ and $q/h = 10$. All decisions are evaluated under the same reinforced environment.

Generator	Disp.	Tail (%)	S	Cost	Error (%)	Fill (%)	Runtime (s)
Independent Poisson	0.99	1.20	6	36.43	13.71	81.49	0.02
Gamma-Poisson/NB	3.95	9.93	8	32.04	0.00	90.88	0.03
Dirichlet-Poisson RCS	4.01	12.28	8	32.04	0.00	90.88	0.03
Finite-memory matrix urn	1.00	1.18	6	36.43	13.71	81.49	0.80

D. Matrix reinforcement and finite memory

Let R be a non-negative replacement matrix with constant row sum. In the symmetric cross-share design,

$$R_{ii} = 1 - \kappa, \qquad R_{ij} = \frac{\kappa}{K-1}, \quad i \neq j.$$

With row-vector urn state $\boldsymbol{b}_t$, period count $\boldsymbol{n}_t$ and memory factor γ, the period update is

$$\boldsymbol{b}_{t+1} = \boldsymbol{a} + \gamma(\boldsymbol{b}_t - \boldsymbol{a}) + \boldsymbol{n}_t R.$$

The baseline vector $\boldsymbol{a}$ anchors the process. The accumulating case is $\gamma = 1$; values below one discount prior reinforcement. Cross-share $\kappa = 0$ reinforces only the observed type, whereas positive κ distributes part of the increment across types. The joint sensitivity grid in Table 5 shows that κ and γ interact; one cannot be interpreted as a simple rescaling of the other.

E. Decision rules

The scalar one-period loss is

$$L(s, d) = h(s - d)_+ + q(d - s)_+.$$

For a known predictive distribution, the optimal continuous critical fractile is $q/(q + h)$; the implementation selects the smallest integer achieving that quantile. Model regret is

$$100 \times \frac{E\{L(S_{\text{model}}, D)\} - E\{L(S_{\text{oracle}}, D)\}}{E\{L(S_{\text{oracle}}, D)\}}.$$

Scarf's moment-robust comparator minimizes the maximum expected loss over distributions with specified mean μ and variance σ^2. For an integer candidate s, the evaluated upper bound is

$$\frac{h-q}{2}(s - \mu) + \frac{h+q}{2}\sqrt{\sigma^2 + (s-\mu)^2}.$$

The can-order simulator applies demand, records unmet demand, charges end-of-period holding, and then replenishes. The independent rule pays $K_f = 18$ for each type ordered. The coordinated rule pays K_f once when any type reaches s and then orders every type at or below c up to S. This difference explains why coordination can reduce cost even when fill rates are nearly equal.

F. Concentration estimation

Given period vectors $\boldsymbol{y}_t$ and fitted baseline shares $\boldsymbol{p}_t$, θ is estimated by maximizing

$$\ell(\theta) = \sum_t \left[\log\Gamma(\theta) - \log\Gamma(\theta + n_t) + \sum_i \{\log\Gamma(\theta p_{ti} + y_{ti}) - \log\Gamma(\theta p_{ti})\}\right].$$

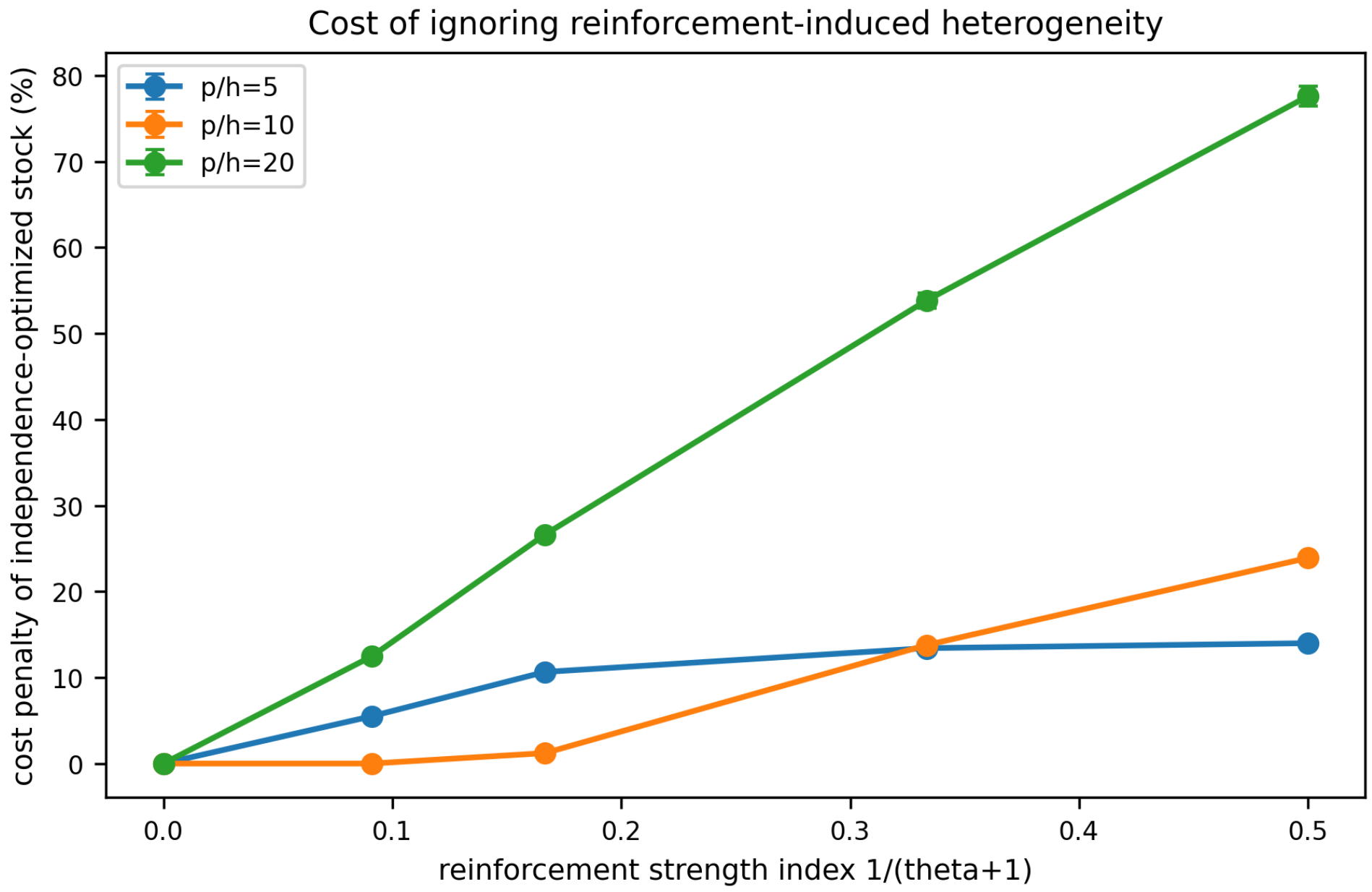

Figure 2: Supplementary scalar independence-calibration experiment. Cost penalties rise when reinforcement is strong and lost events are expensive.

The scalar optimization is carried out on $\log \theta$ over $\log(0.05)$ to $\log(5000)$. When no repeated composition sample exists, the procedure does not estimate θ. It reports a break-even curve over a stated grid and asks whether the decision changes within a plausible range supplied by the analyst.

G. Public-data procedures

RAND-HIE

Health status is formed from the distributed binary indicators: poor takes precedence over fair, fair over good, and observations with no indicator are labeled excellent. The outcome is annual physician visits. Within each of 200 stratified splits, the training data determine means, variances, predictive parameters and integer stock levels. The held-out half supplies all reported costs and fill rates. This is a cross-sectional transfer test and should not be interpreted as a temporal forecast.

NYC 311

The exact fixed query aggregates dataset `erm2-nwe9` by `date_trunc_ymd(created_date)` and complaint type between 1 January 2022 and 1 January 2025 (end exclusive). The five retained categories produce a complete 1,096-day panel. The supplied download script validates all 5,480 category-day cells.

At each rolling origin, five Poisson generalized linear models are fitted on the preceding 365 days:

$$\log \mu_{ti} = \beta_{0i} + \mathrm{DOW}_t + \mathrm{Month}_t + \beta_{1i}\,\mathrm{Trend}_t\,.$$

Negative-binomial dispersion is estimated by residual moments. The composition parameter is estimated from observed daily compositions relative to the fitted calendar shares. Each candidate model chooses daily, category-specific capacity for the next 28 days and is scored on the same observed counts. The temporal drift index is the average absolute difference between observed and predicted total daily volume, divided by average predicted volume.

H. Complete simulation settings

Table 3: Simulation and validation settings.

Experiment	Settings
Classical moments	$K = 4$, $m = 220$, 12,000 paths; $\theta = 1, 2, 5, \infty$; master seed 20260531.
Limiting proportions	20,000 Dirichlet draws per θ.
Scalar generator benchmark	$K = 4$, $\Lambda = 12$, $\theta = 2$, $q/h = 10$, 10,000 environments.
Healthcare stress test	Weekly mean 55 visits per 1,000 patient-years; $q/h = 10$; $\theta = 10, 5, 2, 1$.
Generic independence cost	$\Lambda = 12$, first-type share 0.4, $q/h = 5, 10, 20$, $\theta = 1, 2, 5, 10, \infty$.
RAND held-out	200 deterministic stratified 50/50 splits; $q/h = 5, 10, 20$.
NYC rolling validation	365-day training, 28-day testing, 26 origins; five categories; $q/h = 5, 10$; 6,000 predictive scenarios per test date for simulation-based models.
Empirical NYC diagnostics	Non-overlapping 28-day windows; median dispersion, mean absolute correlation, median 95th-percentile/mean.
Joint matrix sensitivity	$K = 4$, $\Lambda = 12$, $\theta = 3$; 36 $\kappa \times \gamma$ cells; 140 paths of 760 periods; 300-period warm-up.
Multi-type transfer	$K = 4$, $\Lambda = 20$; calibration uses 110 paths of 680 periods; tests use 150 paths of 800 periods; common can-order grid.
Cost uncertainty	150,000 reinforced draws; $\Lambda = 12$, $\theta = 2$, first-type share 0.4; design and true $q/h = 2, 5, 10, 20$.
θ learning	True $\theta = 3, 10, 30$; 14, 28, 56, 112, 224 periods; 60 replications per cell; mean total 100.

I. Supplementary application results

Table 4: Healthcare capacity stress test derived from RAND-HIE group shares and mean counts.

θ	Mean	S_{ind}	S_{RCS}	RCS cost	Ind. cost	Penalty (%)	Fill: RCS/ind. (%)
10	55.00	73	93	57.65	71.35	23.76	96.75/91.18
5	55.00	73	104	73.97	99.80	34.93	95.87/86.48
2	55.00	73	118	100.63	151.55	50.60	93.78/77.93
1	55.00	73	124	121.12	196.50	62.23	91.38/70.50

The healthcare calculation is a synthetic capacity stress test scaled from public count summaries. It is not a hospital scheduling study and does not claim that RAND-HIE visits follow an urn process.

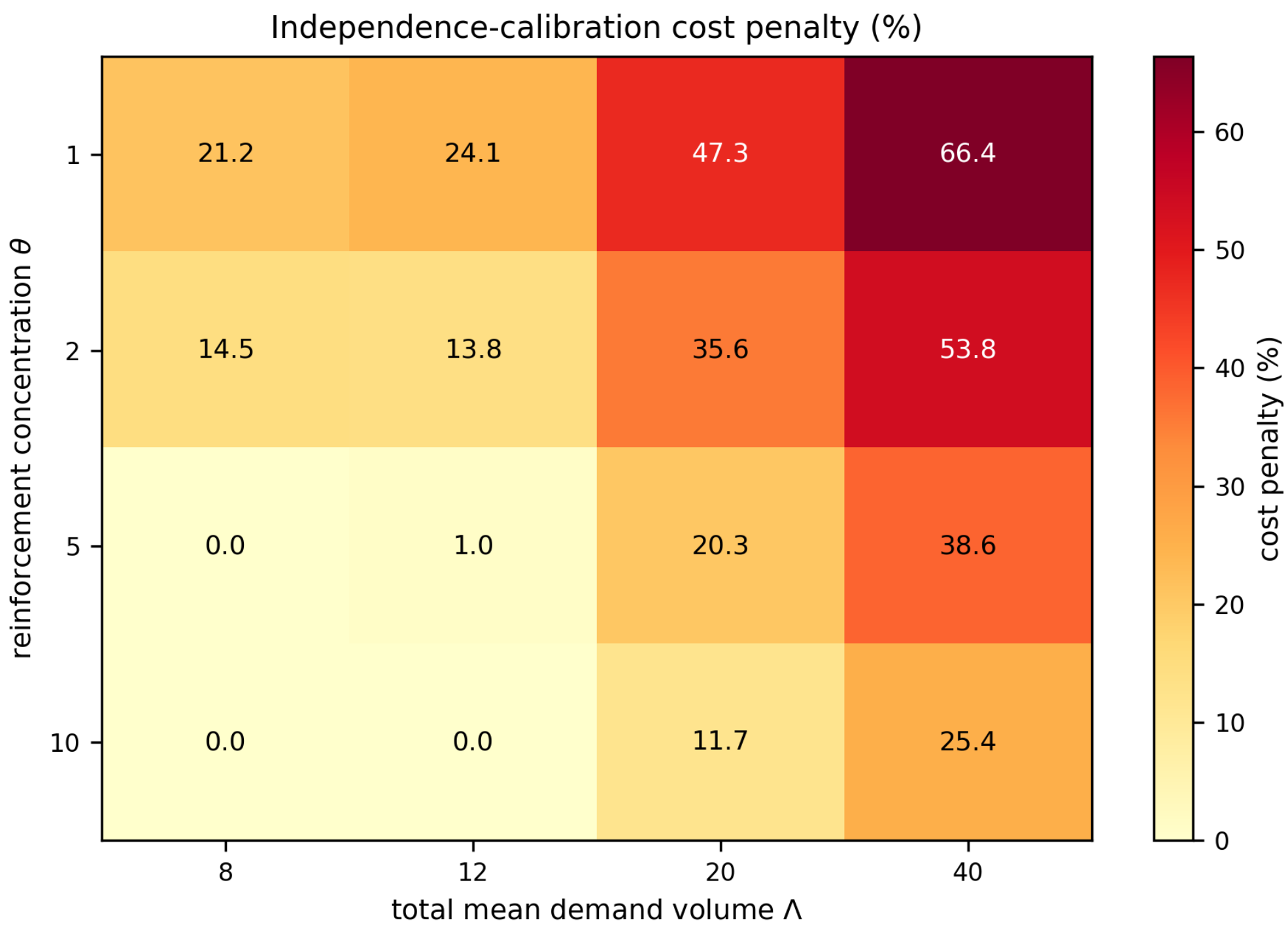

Figure 3: Supplementary sensitivity of the scalar independence-calibration penalty to total demand and reinforcement concentration at $q/h = 10$.

Table 5: Complete joint matrix-urn sensitivity grid. Fill rates are proportions.

κ	γ	Disp.	Lag-1	Pair corr.	Adv. (%)	CI half	Fill ind.	Fill coord.
0.00	0.900	5.037	0.808	-0.266	-3.63	1.19	0.929	0.986
0.10	0.900	1.210	0.225	-0.056	10.43	0.17	0.992	0.995
0.25	0.900	1.048	0.095	-0.015	11.82	0.13	0.994	0.997
0.40	0.900	1.011	0.043	-0.005	12.22	0.15	0.995	0.998
0.60	0.900	1.003	0.013	-0.001	12.46	0.14	0.995	0.998
0.80	0.900	0.998	-0.006	-0.000	12.62	0.14	0.995	0.998
0.00	0.950	2.767	0.644	-0.210	3.09	0.96	0.969	0.972
0.10	0.950	1.104	0.123	-0.031	11.44	0.16	0.994	0.997
0.25	0.950	1.024	0.052	-0.008	12.24	0.14	0.995	0.997
0.40	0.950	1.008	0.018	-0.002	12.36	0.14	0.995	0.998
0.60	0.950	0.999	0.008	0.001	12.59	0.14	0.995	0.998
0.80	0.950	1.000	-0.002	-0.001	12.68	0.14	0.995	0.998
0.00	0.970	2.212	0.553	-0.180	5.16	0.73	0.978	0.981
0.10	0.970	1.070	0.083	-0.019	11.96	0.15	0.994	0.997
0.25	0.970	1.016	0.029	-0.006	12.31	0.13	0.995	0.998
0.40	0.970	1.003	0.013	0.001	12.45	0.12	0.995	0.998
0.60	0.970	1.005	0.010	0.000	12.51	0.13	0.995	0.998
0.80	0.970	0.996	-0.004	-0.002	12.65	0.15	0.995	0.998
0.00	0.985	1.957	0.492	-0.164	6.52	0.64	0.982	0.986
0.10	0.985	1.073	0.079	-0.025	11.67	0.18	0.994	0.997
0.25	0.985	1.011	0.016	-0.002	12.42	0.12	0.995	0.998
0.40	0.985	1.001	0.005	-0.001	12.59	0.12	0.995	0.998
0.60	0.985	1.000	0.003	0.002	12.67	0.13	0.995	0.998
0.80	0.985	1.006	-0.003	0.001	12.74	0.13	0.995	0.998
0.00	0.995	1.931	0.487	-0.161	6.62	0.69	0.982	0.985
0.10	0.995	1.131	0.116	-0.038	11.29	0.22	0.993	0.996
0.25	0.995	1.006	0.012	-0.004	12.49	0.13	0.995	0.998
0.40	0.995	1.000	0.003	-0.002	12.60	0.14	0.995	0.998
0.60	0.995	0.996	0.001	-0.001	12.50	0.14	0.995	0.998
0.80	0.995	1.002	-0.000	0.000	12.68	0.13	0.995	0.998
0.00	1.000	1.976	0.495	-0.163	6.36	0.69	0.982	0.985
0.10	1.000	1.162	0.141	-0.047	11.07	0.23	0.993	0.996
0.25	1.000	1.022	0.020	-0.006	12.42	0.14	0.994	0.998
0.40	1.000	1.003	0.002	-0.001	12.64	0.14	0.995	0.998
0.60	1.000	1.002	-0.005	0.002	12.59	0.13	0.995	0.998
0.80	1.000	0.995	-0.000	0.002	12.63	0.14	0.995	0.998

Table 6: Concentration-parameter learning experiment.

True θ	Periods	Median $\hat{\theta}$	Med. abs. error (%)	P05	P95	Exact (%)	Within 1 (%)
3	14	3.10	13.17	2.07	4.21	58.3	96.7
3	28	2.93	9.17	2.33	3.57	68.3	100.0
3	56	2.95	6.22	2.63	3.49	88.3	100.0
3	112	2.98	4.39	2.74	3.22	98.3	100.0
3	224	3.00	4.12	2.76	3.27	100.0	100.0
10	14	9.83	13.26	7.12	14.05	60.0	100.0
10	28	9.84	8.26	7.84	12.62	66.7	100.0
10	56	10.14	9.08	8.51	12.26	55.0	100.0
10	112	10.18	4.91	9.07	11.38	66.7	100.0
10	224	10.03	3.61	9.19	10.91	78.3	100.0
30	14	30.91	13.59	22.72	49.93	80.0	100.0
30	28	30.85	12.40	23.55	40.07	83.3	100.0
30	56	29.71	10.40	25.80	37.26	93.3	100.0
30	112	30.33	6.09	26.57	35.85	100.0	100.0

True θ	Periods	Median $\hat{\theta}$	Med. abs. error (%)	P05	P95	Exact (%)	Within 1 (%)
30	224	30.22	4.44	27.18	33.31	100.0	100.0

Table 7: Break-even reinforcement grid for the no-sample stress-test mode.

Λ	q/h	Independent S	Break-even θ	Break-even ρ	First changed S
8	2	4	0.428	0.70033	5
8	5	5	4.624	0.17781	6
8	10	6	6.286	0.13725	7
8	20	6	125.540	0.00790	7
12	2	6	0.996	0.50108	7
12	5	7	9.228	0.09777	8
12	10	8	14.627	0.06399	9
12	20	9	18.416	0.05150	10
20	2	9	7.914	0.11218	10
20	5	11	15.794	0.05954	12
20	10	12	36.752	0.02649	13
20	20	13	58.257	0.01688	14
40	2	18	11.618	0.07925	19
40	5	20	62.906	0.01565	21
40	10	21	500.000	0.00200	22
40	20	23	158.057	0.00629	24

Table 8: Cost-ratio uncertainty: regret under reinforced test demand.

True q/h	Design q/h	Poisson regret (%)	RCS regret (%)
2	2	0.00	0.00
2	5	1.61	16.37
2	10	7.25	43.05
2	20	16.37	77.61
5	2	21.06	21.06
5	5	8.70	0.00
5	10	1.94	7.85
5	20	0.00	26.64
10	2	69.60	69.60
10	5	41.10	8.13
10	10	20.99	0.00
10	20	8.13	8.29
20	2	164.34	164.34
20	5	108.78	36.72
20	10	66.87	5.02
20	20	36.72	0.00

J. Reproducibility checks

The reproducibility ZIP has a single top-level folder and uses standard DEFLATE compression. It includes a README, MIT license, pinned requirements, two main analysis scripts, the exact NYC download script, the fixed public aggregate, generated tables and a SHA-256 manifest. The package was reopened after creation, every archived file passed CRC validation, and both analysis scripts completed with seed 20260531. The package does not require Zenodo, GitHub, proprietary data or a proprietary optimizer.
