# Supplementary material for: Reinforced-count simulation for decision calibration under over-dispersed multi-type service demand
Source: PLoS One. 2026 Sep 18;21(9):e0358767. doi: 10.1371/journal.pone.0358767 (PMC13588529; doi:10.1371/journal.pone.0358767)
Supplement: Supplementary file 2 [file pone.0358767.s002.zip › S1_Code/analysis/figures/fig1_public_data_diagnostic.pdf]

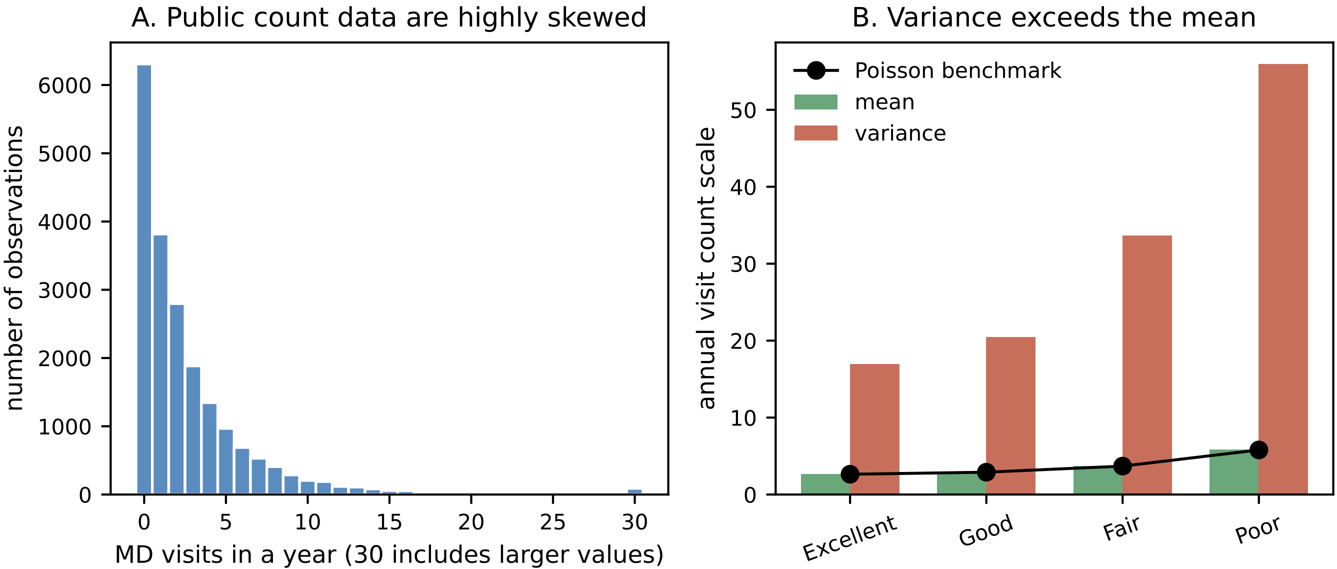

A. Public count data are highly skewed
number of observations
0
1000
2000
3000
4000
5000
6000
0
5
10
15
20
25
30
MD visits in a year (30 includes larger values)
B. Variance exceeds the mean
Poisson benchmark
mean
variance
annual visit count scale
0
10
20
30
40
50
Excellent
Good
Fair
Poor
