# Supplementary material for: Reinforced-count simulation for decision calibration under over-dispersed multi-type service demand
Source: PLoS One. 2026 Sep 18;21(9):e0358767. doi: 10.1371/journal.pone.0358767 (PMC13588529; doi:10.1371/journal.pone.0358767)
Supplement: Supplementary file 2 [file pone.0358767.s002.zip › S1_Code/analysis/figures/fig1_workflow.pdf]

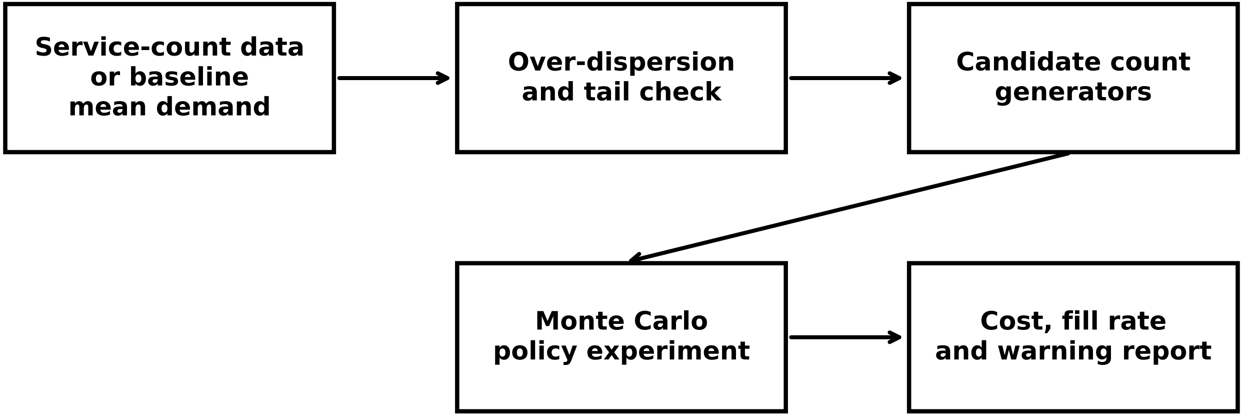

Benchmark models: independent Poisson, gamma-Poisson/negative binomial, reinforced urn, finite-memory matrix urn

Decision outputs: selected capacity/stock level, expected cost, fill rate, tail exposure and calibration warning
