# Supplementary material for: Reinforced-count simulation for decision calibration under over-dispersed multi-type service demand
Source: PLoS One. 2026 Sep 18;21(9):e0358767. doi: 10.1371/journal.pone.0358767 (PMC13588529; doi:10.1371/journal.pone.0358767)
Supplement: Supplementary file 2 [file pone.0358767.s002.zip › S1_Code/analysis/figures/fig2_classical_diagnostics.pdf]

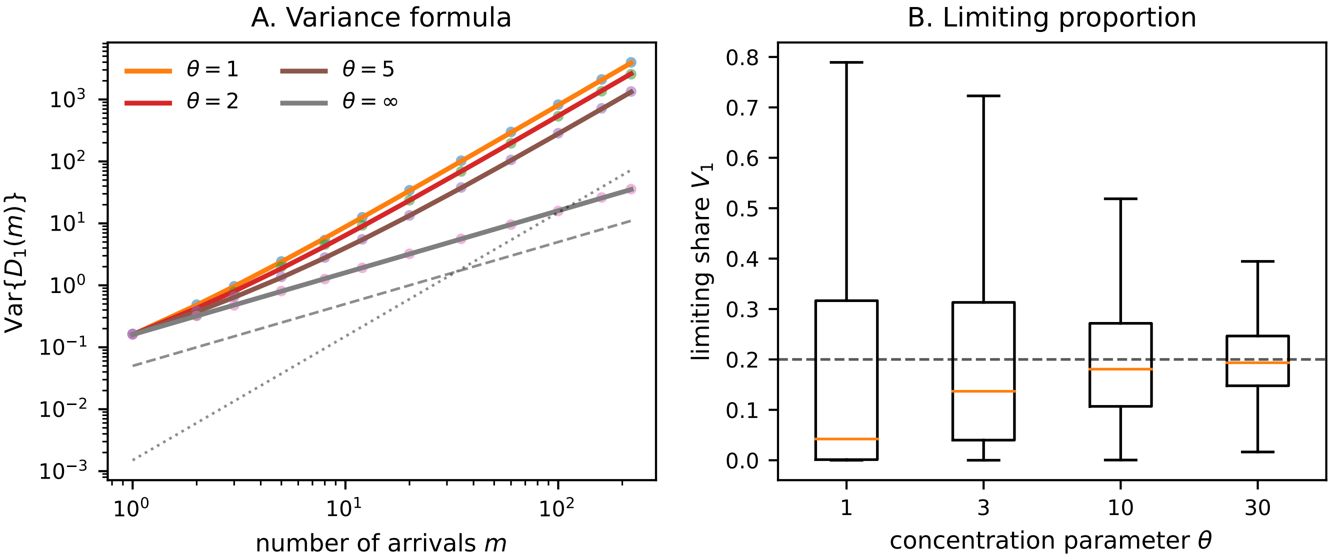
A. Variance formula
B. Limiting proportion
$\theta = 1$
$\theta = 2$
$\theta = 5$
$\theta = \infty$
$\mathrm{Var}\{D_1(m)\}$
number of arrivals $m$
$10^{-3}$
$10^{-2}$
$10^{-1}$
$10^{0}$
$10^{1}$
$10^{2}$
$10^{3}$
limiting share $V_1$
0.0
0.1
0.2
0.3
0.4
0.5
0.6
0.7
0.8
1
3
10
30
concentration parameter $\theta$
