# Supplementary material for: Reinforced-count simulation for decision calibration under over-dispersed multi-type service demand
Source: PLoS One. 2026 Sep 18;21(9):e0358767. doi: 10.1371/journal.pone.0358767 (PMC13588529; doi:10.1371/journal.pone.0358767)
Supplement: Supplementary file 2 [file pone.0358767.s002.zip › S1_Code/analysis/figures/fig3_cost_penalty.pdf]

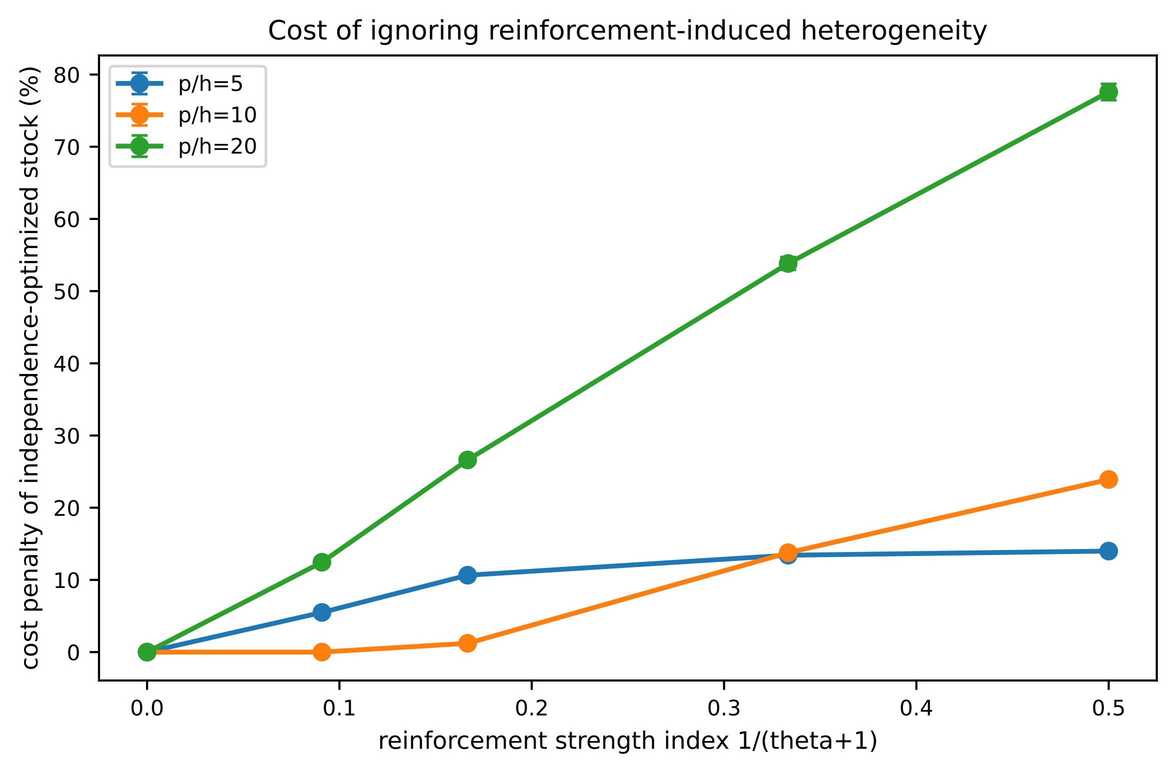

Cost of ignoring reinforcement-induced heterogeneity
cost penalty of independence-optimized stock (%)
reinforcement strength index 1/(theta+1)
p/h=5
p/h=10
p/h=20
0
10
20
30
40
50
60
70
80
0.0
0.1
0.2
0.3
0.4
0.5
