# Supplementary material for: Reinforced-count simulation for decision calibration under over-dispersed multi-type service demand
Source: PLoS One. 2026 Sep 18;21(9):e0358767. doi: 10.1371/journal.pone.0358767 (PMC13588529; doi:10.1371/journal.pone.0358767)
Supplement: Supplementary file 2 [file pone.0358767.s002.zip › S1_Code/analysis/figures/fig4_cross_share_coordination.pdf]

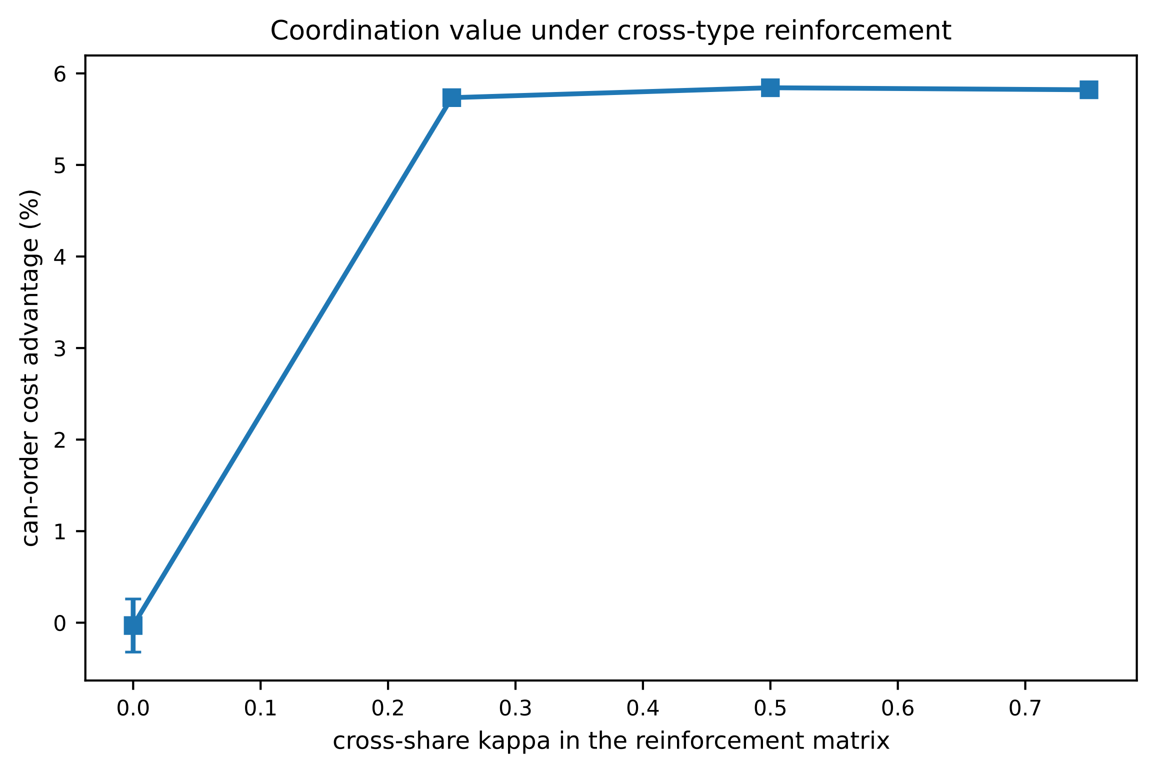

Coordination value under cross-type reinforcement
can-order cost advantage (%)
cross-share kappa in the reinforcement matrix
0
1
2
3
4
5
6
0.0
0.1
0.2
0.3
0.4
0.5
0.6
0.7
