# Supplementary material for: Reinforced-count simulation for decision calibration under over-dispersed multi-type service demand
Source: PLoS One. 2026 Sep 18;21(9):e0358767. doi: 10.1371/journal.pone.0358767 (PMC13588529; doi:10.1371/journal.pone.0358767)
Supplement: Supplementary file 2 [file pone.0358767.s002.zip › S1_Code/analysis/figures/fig5_forgetting_sensitivity.pdf]

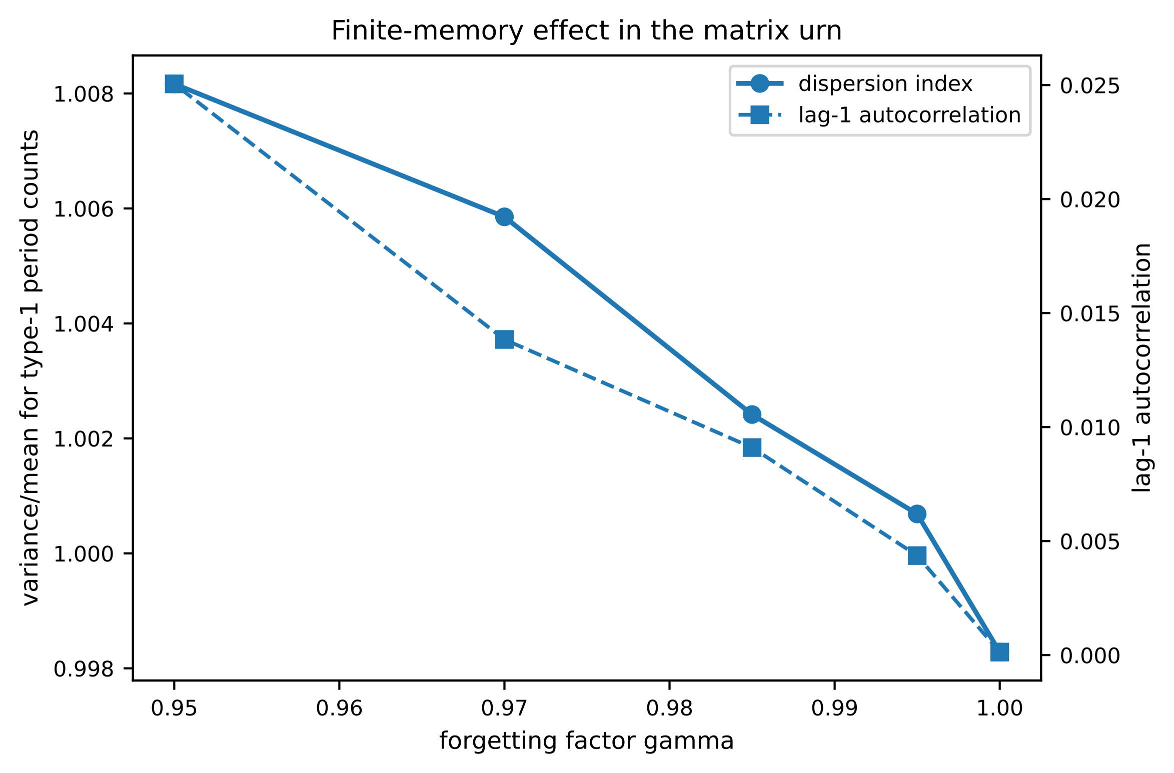

Finite-memory effect in the matrix urn
dispersion index
lag-1 autocorrelation
variance/mean for type-1 period counts
lag-1 autocorrelation
forgetting factor gamma
1.008
1.006
1.004
1.002
1.000
0.998
0.025
0.020
0.015
0.010
0.005
0.000
0.95
0.96
0.97
0.98
0.99
1.00
