# Supplementary material for: Reinforced-count simulation for decision calibration under over-dispersed multi-type service demand
Source: PLoS One. 2026 Sep 18;21(9):e0358767. doi: 10.1371/journal.pone.0358767 (PMC13588529; doi:10.1371/journal.pone.0358767)
Supplement: Supplementary file 2 [file pone.0358767.s002.zip › S1_Code/analysis/figures/fig7_sensitivity_heatmap.pdf]

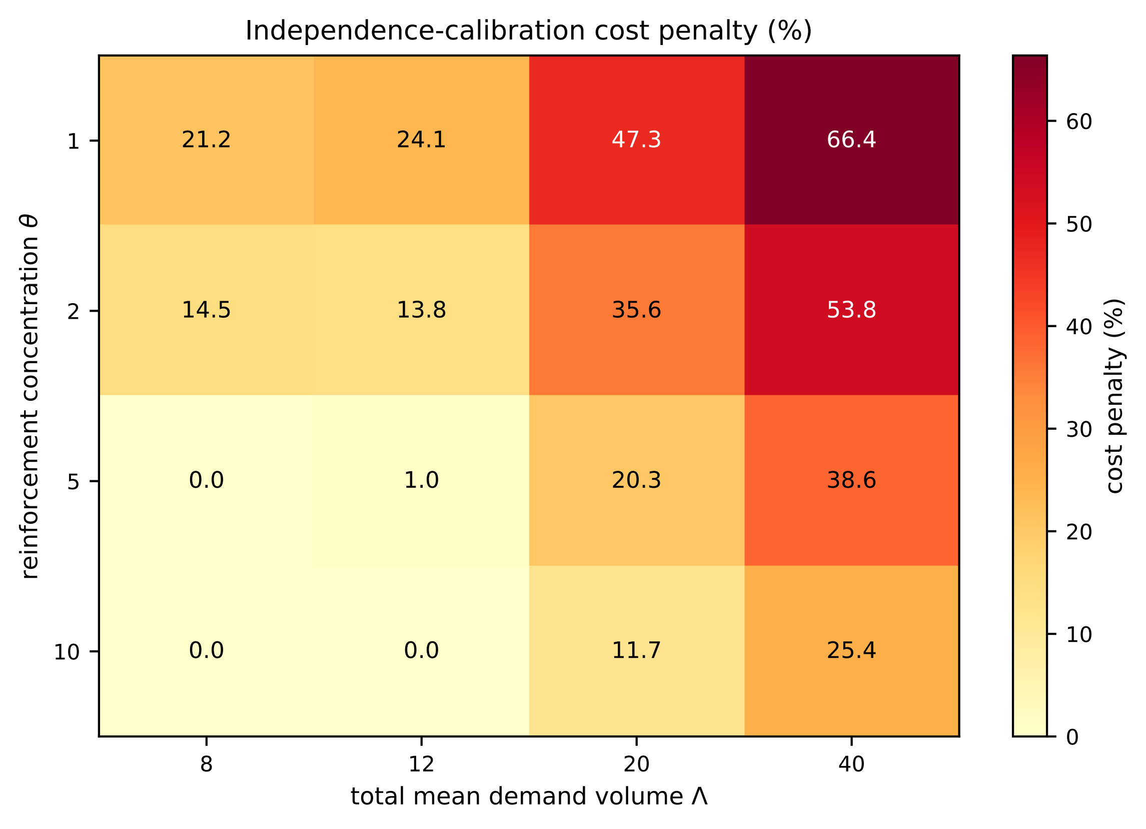

Independence-calibration cost penalty (%)
reinforcement concentration θ
1
2
5
10
total mean demand volume Λ
8
12
20
40
21.2
24.1
47.3
66.4
14.5
13.8
35.6
53.8
0.0
1.0
20.3
38.6
0.0
0.0
11.7
25.4
cost penalty (%)
0
10
20
30
40
50
60
