# Supplementary material for: Reinforced-count simulation for decision calibration under over-dispersed multi-type service demand
Source: PLoS One. 2026 Sep 18;21(9):e0358767. doi: 10.1371/journal.pone.0358767 (PMC13588529; doi:10.1371/journal.pone.0358767)
Supplement: Supplementary file 2 [file pone.0358767.s002.zip › S1_Code/analysis/figures/fig8_oos_validation.pdf]

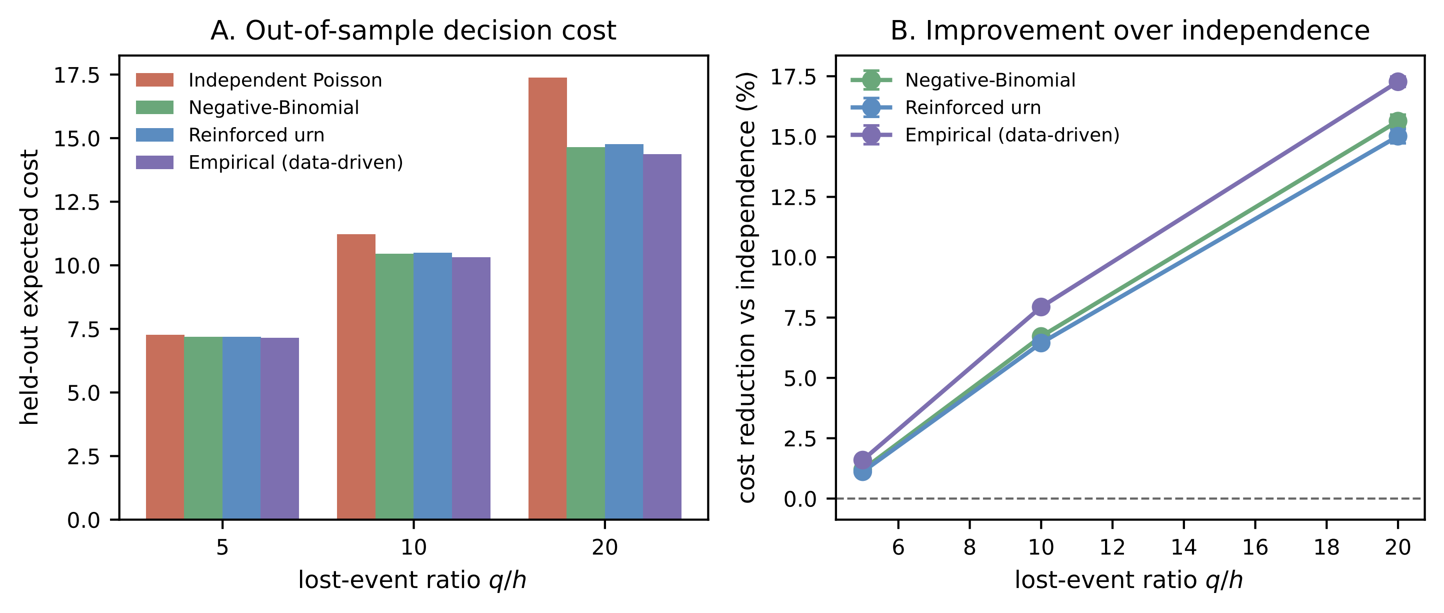

A. Out-of-sample decision cost
held-out expected cost
Independent Poisson
Negative-Binomial
Reinforced urn
Empirical (data-driven)
0.0
2.5
5.0
7.5
10.0
12.5
15.0
17.5
5
10
20
lost-event ratio q/h
B. Improvement over independence
cost reduction vs independence (%)
Negative-Binomial
Reinforced urn
Empirical (data-driven)
0.0
2.5
5.0
7.5
10.0
12.5
15.0
17.5
6
8
10
12
14
16
18
20
lost-event ratio q/h
