# Supplementary material for: Reinforced-count simulation for decision calibration under over-dispersed multi-type service demand
Source: PLoS One. 2026 Sep 18;21(9):e0358767. doi: 10.1371/journal.pone.0358767 (PMC13588529; doi:10.1371/journal.pone.0358767)
Supplement: Supplementary file 2 [file pone.0358767.s002.zip › S1_Code/analysis/figures/fig9_oos_fill.pdf]

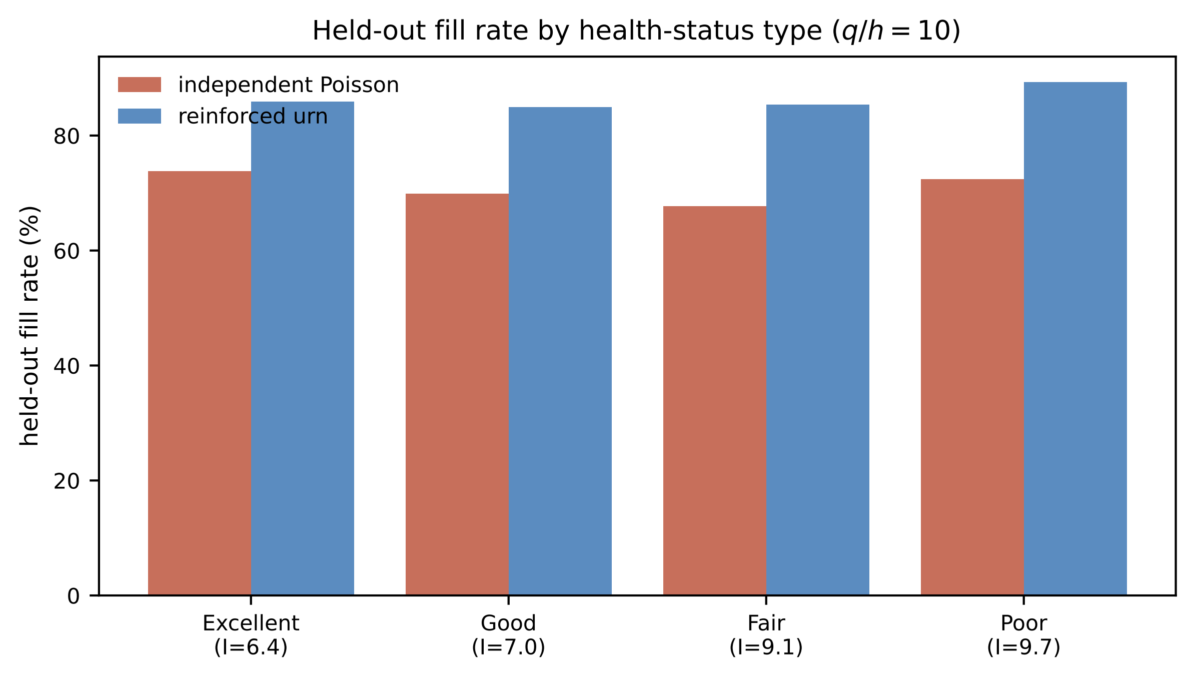

Held-out fill rate by health-status type ($q/h = 10$)
independent Poisson
reinforced urn
held-out fill rate (%)
0
20
40
60
80
Excellent (I=6.4)
Good (I=7.0)
Fair (I=9.1)
Poor (I=9.7)
