# Supplementary material for: Reinforced-count simulation for decision calibration under over-dispersed multi-type service demand
Source: PLoS One. 2026 Sep 18;21(9):e0358767. doi: 10.1371/journal.pone.0358767 (PMC13588529; doi:10.1371/journal.pone.0358767)
Supplement: Supplementary file 2 [file pone.0358767.s002.zip › S1_Code/analysis/figures/graphical_abstract.pdf]

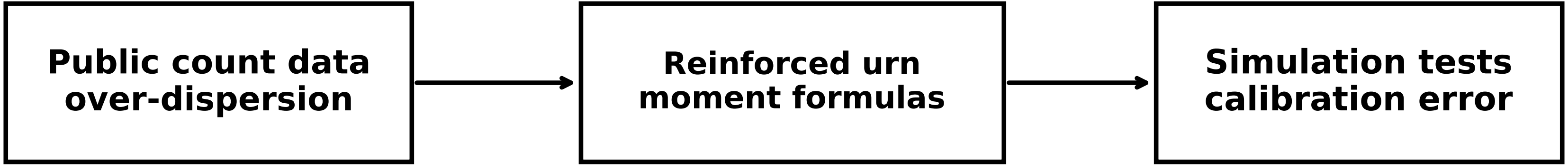

Public count diagnostics motivate reinforced event-count modeling;
Dirichlet-multinomial formulas explain variance inflation and correlation invariance.

Public data -> classical urn moments -> Monte Carlo policy diagnostics
